# Structure of *Escherichia coli* O157:H7 bacteriophage CBA120 tailspike protein 4 baseplate anchor and tailspike assembly domains (TSP4-N)

**DOI:** 10.1038/s41598-022-06073-2

**Published:** 2022-02-08

**Authors:** Kinlin L. Chao, Xiaoran Shang, Julia Greenfield, Sara B. Linden, Adit B. Alreja, Daniel C. Nelson, Osnat Herzberg

**Affiliations:** 1 Institute for Bioscience and Biotechnology Research, University of Maryland, 9600 Gudelsky Drive, Rockville, MD 20850 USA; 2 Department of Chemistry and Biochemistry, University of Maryland, Chemistry Building, 8051 Regents Drive, College Park, MD 20742 USA; 3 Department of Veterinary Medicine, University of Maryland, 8075 Greenmead Drive, College Park, MD 20742 USA

**Keywords:** Molecular biophysics, Structural biology, Molecular modelling, X-ray crystallography, Viral proteins

## Abstract

Four tailspike proteins (TSP1-4) of *Escherichia coli* O157:H7 bacteriophage CBA120 enable infection of multiple hosts. They form a branched complex that attaches to the tail baseplate. Each TSP recognizes a different lipopolysaccharide on the membrane of a different bacterial host. The 335 N-terminal residues of TSP4 promote the assembly of the TSP complex and anchor it to the tail baseplate. The crystal structure of TSP4-N_335_ reveals a trimeric protein comprising four domains. The baseplate anchor domain (AD) contains an intertwined triple-stranded β-helix. The ensuing XD1, XD2 and XD3 β-sheet containing domains mediate the binding of TSP1-3 to TSP4. Each of the XD domains adopts the same fold as the respective XD domains of bacteriophage T4 gp10 baseplate protein, known to engage in protein–protein interactions via its XD2 and XD3 domains. The structural similarity suggests that XD2 and XD3 of TSP4 also function in protein–protein interactions. Analytical ultracentrifugation analyses of TSP4-N_335_ and of domain deletion proteins showed how TSP4-N_335_ promotes the formation of the TSP quaternary complex. TSP1 and TSP2 bind directly to TSP4 whereas TSP3 binding requires a pre-formed TSP4-N_335_:TSP2 complex. A 3-dimensional model of the bacteriophage CBA120 TSP complex has been developed based on the structural and ultracentrifuge information.

## Introduction

Bacteriophage (phage) vB_EcoM_CBA120, discovered by Kutter and colleagues^1^, is a member of the recently defined *Kuttervirus* genus of contractile tailed phages belonging to the *Ackermannviridae* family. The genomes of *Kuttervirus* phages encode up to four tailspike proteins (TSP1-4) that catalyze the breakdown of bacterial host lipopolysaccharide (LPS) substrates. Phage CBA120 ORFs 210–213 encode TSP1-4, respectively. Initially identified as a phage that infects *Escherichia coli* O157:H7^1^, phage CBA120 has now been shown to infect *E. coli* O77, O78 and *Salmonella enterica* serovar Minnesota by utilizing different TSPs^2^. TSP2, TSP3 and TSP4 cleave the O157, O77 and O78 lipopolysaccharide O-antigens, respectively. TSP1 has been postulated to be responsible for the *S. enterica* infection although its polysaccharide target has not been identified. Negative-stained electron micrographs of phage CBA120 showed branched appendages emanating from the contractile tail’s baseplate^3^, which were attributed to the quaternary complex formed by TSP1-4.

**Figure 1:**
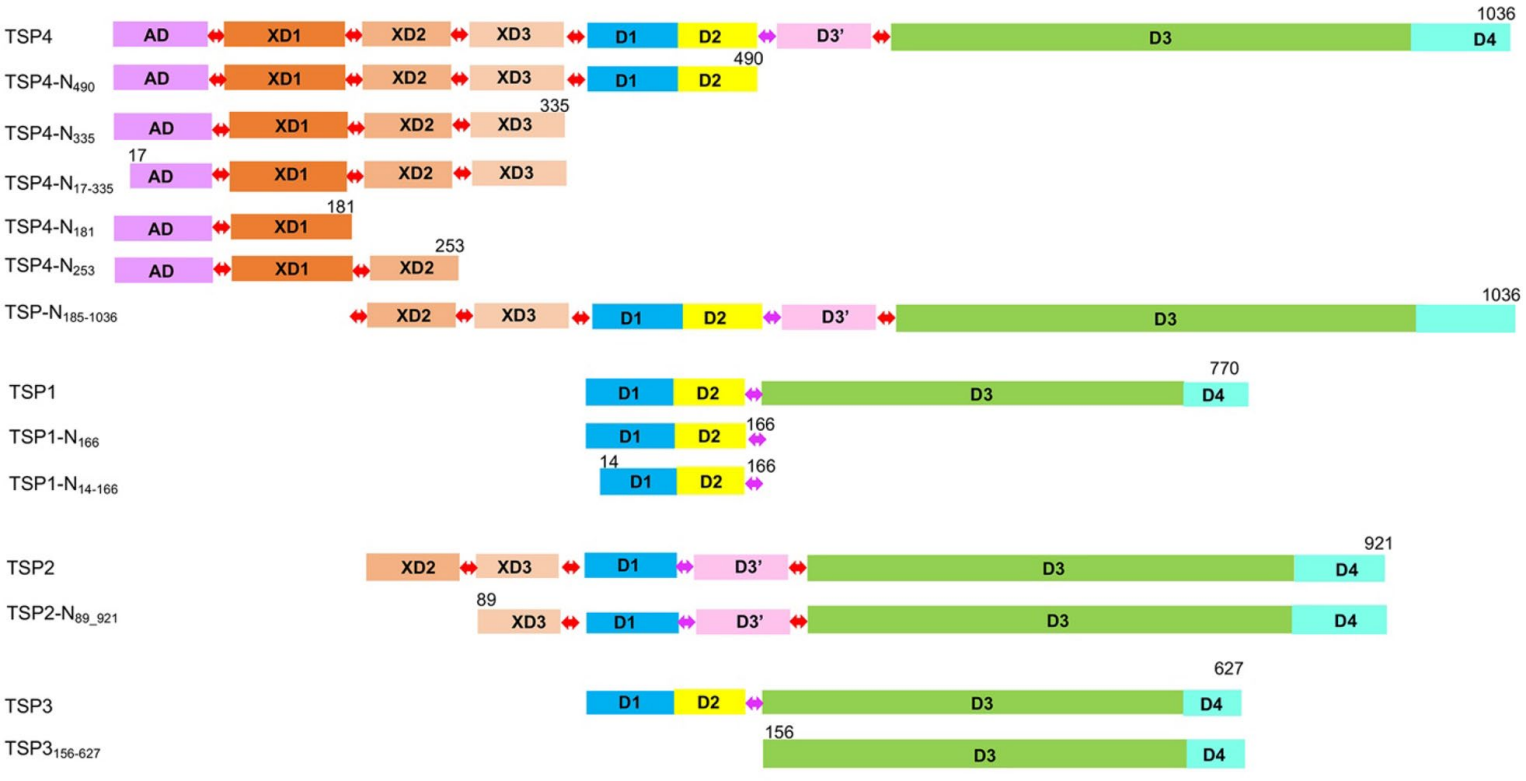
Domain organization of phage CBA120 TSP1-4 and recombinant TSP fragments. The N-terminal baseplate anchor domain (AD) is colored magenta. Three T4 gp10-like domains of TSP4 (XD1, XD2 and XD3) and two of TSP2 (XD2 and XD3) are colored different shades of brown. Domain linkers are indicated as red double arrows. The helical neck separating the head region from the TSP body/catalytic domains is indicated as magenta double arrows. The head domains, D1 and D2, are colored blue and yellow, respectively. The D3’ domain inserted after the neck in TSP2 and TSP4 is colored pink. The β-helix domain, D3, and the C-terminal domain, D4, are colored green and cyan, respectively. Structures of the D1-D4 regions have been determined for all four TSPs^2,4–6^. The AD-XD1-XD2-XD3 region is the focus of the current study.

TSP1-4 are multidomain trimeric proteins^2,4–6^. Each TSP contains a trimeric β-helix domain, D3, with enzymatic activity for the cleavage of a LPS glycosidic bond, followed by a C-terminal domain, D4, of unknown function (Fig. 1). Each TSP trimer contains three catalytic sites, located at the interfaces between D3 subunits, as verified by site-directed mutagenesis studies^5,6^. Preceding the D3 N-terminus, phage CBA120’s TSP1, TSP3 and TSP4 contain two domains (D1 and D2) homologous in sequence and structure, whereas TSP2 carries only the D1 domain. The D1-D2 and D3-D4 regions are designated the “head” and “body”, respectively (Fig. 1). A short α-helical “neck” connects the TSP head and body. TSP2 and TSP4 contain N-terminal regions preceding their head domains, ~ 170 and ~ 340 amino acid residues long, respectively, and an additional small domain following the neck, D3’ (Fig. 1). Plattner et al. used Hidden Markov Model (HMM) analyses to identify four domains (termed herewith AD, XD1, XD2 and XD3) spanning the ~ 340-residue N-terminal region of TSP4, whereas TSP2 N-terminal region contains only the XD2 and XD3 domains^2^. They also found that the three TSP4 XD domains are structurally related to the respective domains of the baseplate protein gp10 from bacteriophage T4^7^. In addition, the gp66 TSP from the *E. coli* bacteriophage G7C contains a N-terminal region that attaches to the baseplate, followed by XD2 and XD3 domains. Based on this homology between CBA120 TSP4 and gp66 TSP, Plattner and colleagues inferred that the eighty N-terminal amino acid residues of TSP4 bind to the tail baseplate of phage CBA120 even though the N-terminal regions of gp66 and TSP4 lack sequence homology. We refer to this baseplate anchor domain as AD (Fig. 1).

Negative-staining electron microscopy (EM) of CBA120 TSP ternary and quaternary complexes^2^ revealed branched structures that resemble the tail appendages seen in the EM images of the intact phage^1^. Based on the EM and size-exclusion chromatography (SEC) analyses, Plattner and colleagues concluded that the TSP2 and TSP4 complex must form first to enable subsequent attachments of TSP1 and TSP3. They hypothesized that the N-terminal domains of TSP2 and TSP4 mediate protein–protein interactions that give rise to the branched appendages emanating from the tail baseplate. The crystal structures of the D1-D4 domains of all four phage CBA120 TSPs have been determined^2,4–6^. However, no structure has been reported for the N-terminal regions of either TSP2 or TSP4. Here, we report the crystal structure of the 335-residue N-terminal TSP4 region (TSP4-N_335_) that contains the assembly sites for TSP1-3 as well as the baseplate anchor site. We use analytical ultracentrifugation (AUC) studies to characterize the interactions of TSP4-N with TSP1, TSP2 and TSP3. The emerging model serves as a paradigm for the branched assemblies of TSPs from other *Kuttervirus* genus members.

## Results and discussion

### Structure determination

The boundaries of phage CBA120 TSP4-N proteins were chosen based on the predicted locations of linkers between domains. The genes were synthesized, sub-cloned, produced in *E. coli*, purified and crystallized as detailed in the methods section. TSP4-N_335_ crystals grown in KNa-tartrate solution diffracted to the highest resolution. However, the SeMet TSP4-N_335_ containing three constitutive methionines and three additional engineered methionines designed to increase the anomalous signal (Leu12Met, Ile31Met and Leu145Met), were all twinned when grown in KNa-tartrate solution. Thus, phase determination was performed with SeMet protein crystals grown from solutions containing lithium sulfate (Table 1). The diffraction quality was rather poor and required five merged data sets to yield sufficient anomalous diffraction signal for the identification of 15 Se sites, corresponding to 5 SeMet residues per monomer. Phase determination by the SAD method yielded an electron density map that enabled the building of the AD-XD1-XD2 trimer. However, the electron density associated with the XD3 domains could not be traced. Subsequently, the three resolved SeMet-TSP4-N_335_ domains were used as search models for molecular replacement with diffraction data from the wild-type protein crystals grown in KNa-tartrate. The resulting electron density map enabled tracing of the XD3 domains. This crystal form contains two trimers in the asymmetric unit (Table 1), however, only one XD3 domain could be modeled (Molecule A in the coordinates deposited in the Protein Data Bank (PDB), accession number 7REJ). This XD3 domain structure enabled the placement and refinement of the three XD3 domains in the SeMet protein crystal asymmetric unit.

**Table 1:** TSP4-N data collection and refinement statistics.

Protein Precipitant	TSP4-N_335_ SeMet LiSO_4_	TSP4-N_335_ tartrate	TSP4-N_250_ PEG8000
**Data collection**			
No. merged data sets	5	1	1
Space group (number)	P4_1_2_1_2 (92)	R3 (146)	R32 (155)
Cell dimension (Å)	a = b = 67.9, c = 607.2	a = b = 78.0, c = 326.9	A = b = 84.7, c = 225.1
Wavelength (Å)	0.9793	1.0332	1.0332
Resolution (Å)	3.0	2.5	2.8
No. molecules in the ASU	3 (trimer)	2	1
No. observed reflections	1,844,233	66,423	36,002
No. unique reflections	28,173	25,427	7,883
Completeness (%)^a^	94.4 (67.6)	98.9 (98.8)	99.0(97.2)
Multiplicity	66.9	2.6	4.6
*R*_merge_^b^	0.228 (2.43)	0.090 (0.86)	0.070(3.36)
<*I*/σ*I*>	20.4 (0.8)	5.1 (0.9)	7.6(0.2)
Wilson B/anisotropic ΔB (Å^2^)	119.4/33.9	52.0/5.3	138.0/77.8
**SAD phasing and automatic chain building**			
Δanomalous cc1/2	0.907		
No. found/true Se sites	17/15		
No. residues/fragments built	546/44		
Model-map correlation	0.64		
*R*^c^/*R*_free_^d^	0.39/0.43		
**Refinement**			
Resolution range (Å)	30.0–3.0	20.0–2.6	30–3.2
Total no. of reflections	26,761	21,436	5125
Completeness (%)	94.4	98.7	98.9
*R*^c^/*R*_free_^d^	0.225/0.296	0.206/0.229	0.201/0.301
No. protein residues	983	576	241
No. waters/imidazoles	–	32/2	–
RMSD from ideal geometry			
Bond length (Å)/angles (°)	0.019/1.8	0.012/1.5	0.005/1.8
Average B (Å^2^)	141.9 ^e^	80.3 ^e^	151.6
Ramachandran plot (%)			
Allowed/outliers	97.2/2.8	99.8/0.2	99.0/1.0

^a^The values in parentheses are for the highest resolution shells.

^b^*R*_*merge*_ = *Σ*_*hkl*_ [(Σ_*j*_|*I*_*j*_ − < *I* >|)/Σ_*j*_|*I*_*j*_|].

^c^*R* = Σ_*hkl*_||*F*_*o*_| − |*F*_*c*_||/Σ_*hkl*_|*F*_*o*_|, where *F*_*o*_ and *F*_*c*_ are the observed and calculated structure factors, respectively.

^d^*R*_*free*_ is computed from randomly selected 5% of reflections omitted from the refinement.

^e^B factors calculated after TLS refinements (5 and 7 groups for the WT/tartrate and SeMet/LiSO_4_ crystals, respectively).

**Figure 2:**
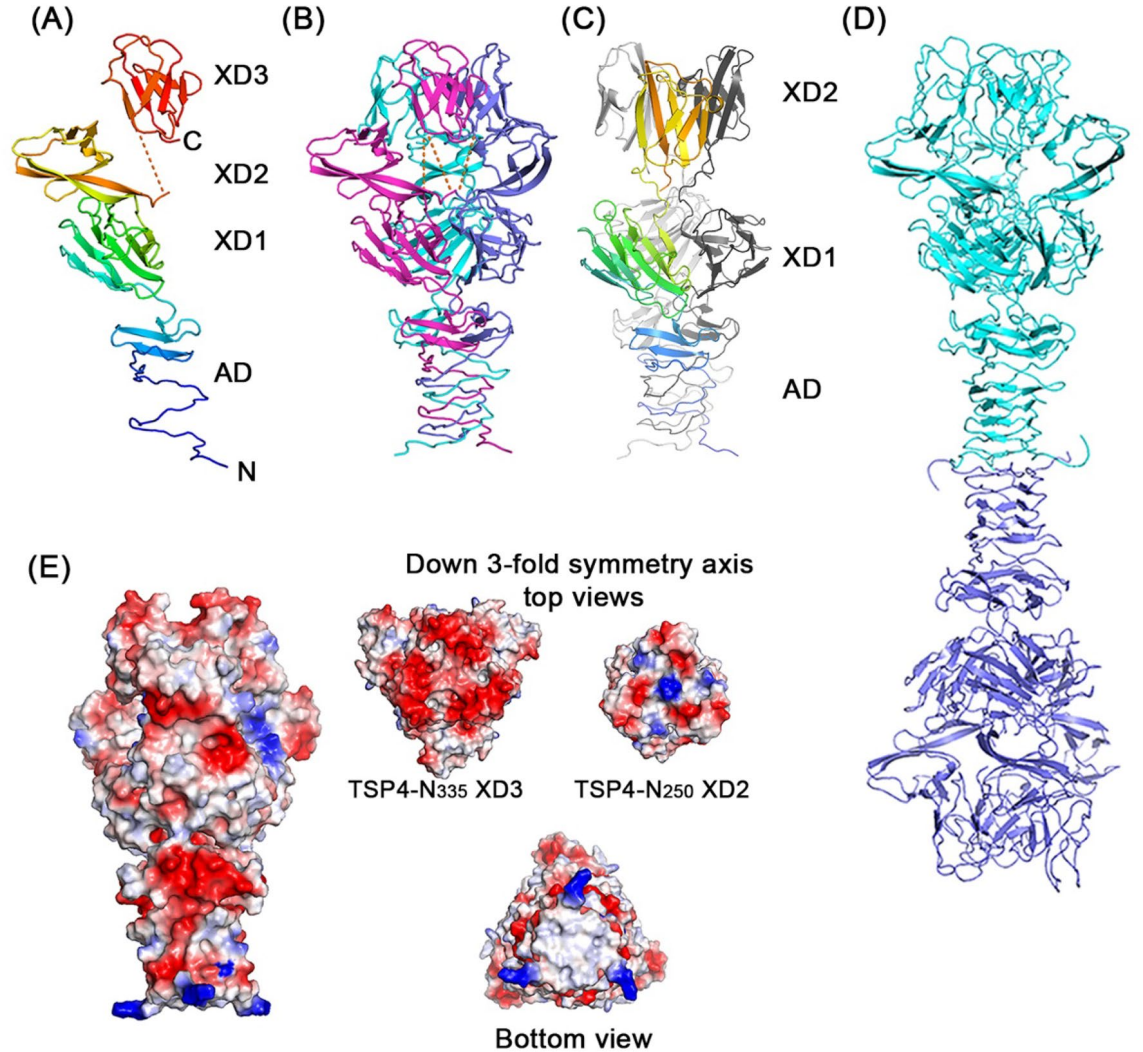
Structures of TSP4-N335 and TSP4-N250. A cartoon representation of (**A**) TSP4-N335 monomer shown with spectrum color from blue N-terminus to red C-terminus. (**B**) TSP4-N335 trimer highlighting the three monomers in different colors. (**C**) TSP4-N250 trimer shown with one subunit in spectrum colors and two subunits in gray. The XD3 domain is missing. The spectrum color range span rainbow colors from blue to orange, to highlight the difference in the XD2 domain locations compared with that seen in TSP4-N335 as shown in (**A**). (**D**) Dimer of TSP4-N335 trimers. The two trimers associate via their N-terminal surfaces perpendicular to a shared threefold symmetry axis of the triple-stranded α-helix. (**E**) Left: Surface vacuum electrostatic potential calculated using PyMol with red color depicting negatively charged regions and blue color depicting positively charged regions. The trimer is viewed along the threefold symmetry axis. Right: Three views down the threefold symmetry axis. The top images show the XD3 surface on the left (based on the TSP4-N335 structure) and the XD2 surface on the right (based on the and TSP4-N250 structure that lacks XD3). The bottom image shows the N-terminal hydrophobic patch (white color) of the AD domain that mediates protein–protein interaction. Side chains that were not associated with electron densities during the refinement were omitted from the experimental structures. However, to fully account for all the charges and dipoles that contribute to the electrostatic potential, these side chains were added with favorable conformations.

A construct comprising the AD-XD1-XD2-XD3 region followed by the D1-D2 head region facilitated AUC studies to examine whether the presence of the head region alters protein–protein interactions. Herewith we refer to this construct as TSP4-N_490_. This protein degraded within the period of crystal growth in poly ethylene glycol 8000 solution (see methods), yielding a structure that lacked the XD3 domain. Although the exact cleavage site is unknown, 250 residues could be traced in the electron density map. In the following discussion, this structure is named TSP4-N_250_ to distinguish it from TSP4-N_253_, one of the protein variants that was designed to identify the TSP4-N domain interactions with partner TSPs by AUC (Fig. 1). The TSP4-N_250_ structure was determined by molecular replacement (Table 1). The search yielded a clear solution that showed three XD2 domain pack together in contrast to the separated XD2 domains seen in the two crystal structures of TSP4-N_335_ (Fig. 2).

### TSP4-N_335_ structure

TSP4-N_335_ associates into an elongated trimer, ~ 115 Å in length and ~ 65 Å at its widest region (Fig. 2). A monomeric subunit comprises four domains; AD, XD1, XD2 and XD3 (Fig. 2A). Residues 7–42 of the AD domains of the three subunits form an intertwined triple-stranded β-helix, a fold previously seen in viral and phage proteins^8^. The 2-turn TSP4 triple-stranded β-helix has a triangular cross section with ~ 18–20 Å long edges. The three polypeptide chains then disengage from the intertwining and each subunit forms a 3-stranded anti-parallel β-sheet. The 3-stranded β-sheets of the trimer subunits associate to form a triangular β-prism II structure along the same threefold symmetry axis as that of the triple-stranded β-helix (Fig. 2B). The triangular cross section of AD at the β-prism II increases to ~ 30 Å. The combination of triple-stranded β-helices and triple β-prism II folds occurs in other bacteriophage proteins, for example, the endosialidase of bacteriophage K1F and the tail fiber gp34 of bacteriophage T4 and^9,10^.

**Figure 3:**
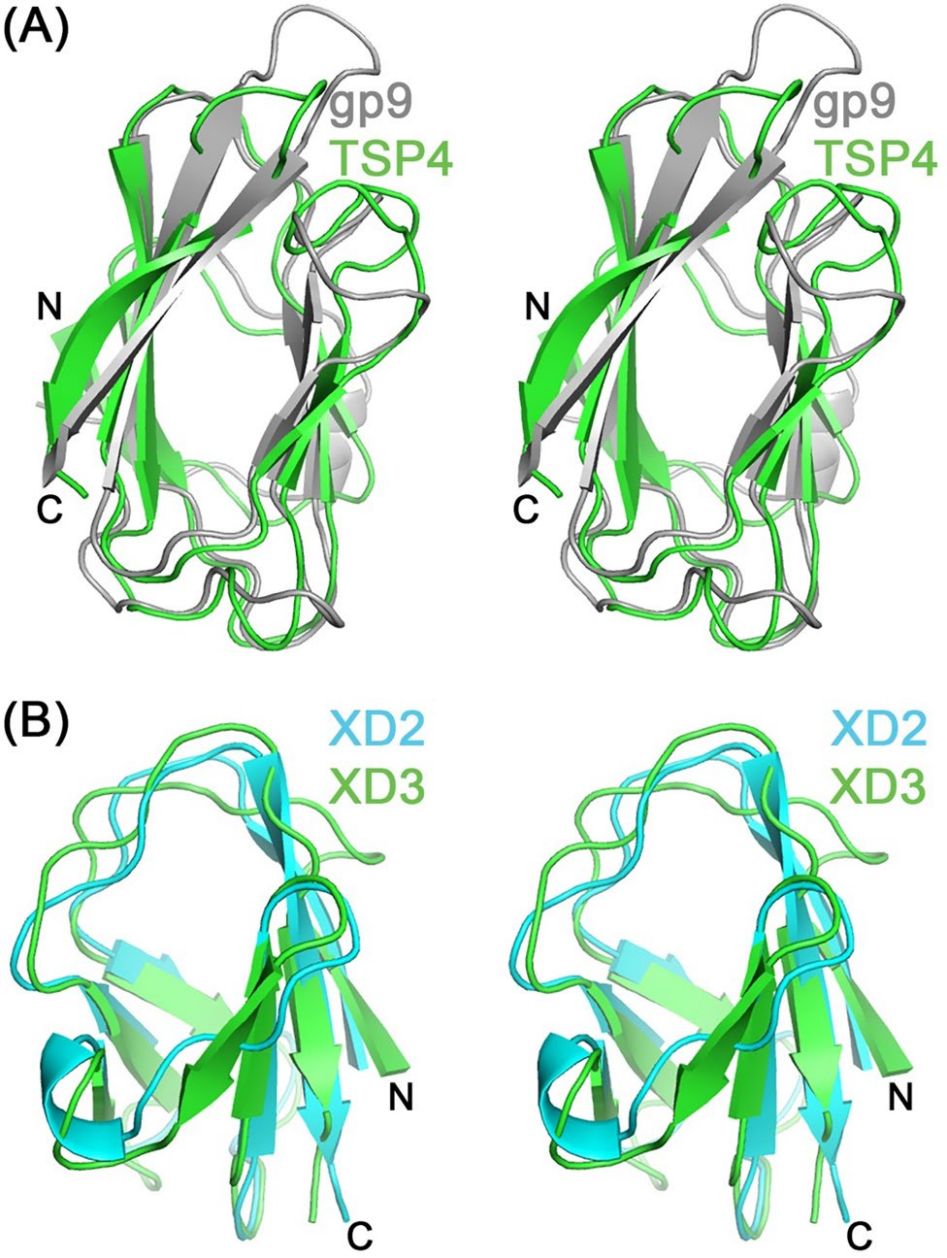
Stereoscopic representation of structural similarity of XD domains. (**A**) Superposition of the XD1 domains of TSP4-N_335_ (green) and phage T4 gp9 protein (gray). (**B**) Superposition of the TSP4-N_335_ XD2 (blue) and XD3 (green) domains.

The TSP4 XD1 domain (amino acid residues 80–178) forms a 9-stranded mixed β-sandwich comprising four β-stranded and five β-stranded β-sheets (Fig. 2). The first 6 β-strands alternate from one β-sheet to the other to form 3 parallel β-strands per sheet. The last 2 β-strands of the 5-stranded β-sheet run antiparallel as does the last β-strand of the 4-stranded β-sheet. The TSP4 XD1 core contains primarily hydrophobic residues. The three XD1 domains of the TSP4 trimer employ both hydrophobic and hydrophilic intermolecular interactions to pack around the same threefold symmetry axis employed by the three AD domains. The DALI structure homology analysis^11^ revealed a phage T4 baseplate gp9 domain as the closest structure homologue of TSP4 XD1 domain^12^ with a high Z score of 12.5 (Fig. 3, Table 2). In addition, the gp10 protein from bacteriophage T4 also contains a XD1 domain^7,13^, albeit with a lower Z score of 5.0 (Table 2). T4 gp10 plays a critical role in the assembly of the tail wedge complex by bindings to protein partners^14^. Interestingly, two bacterial virulence factors contain glycan-binding domains that adopt the same fold; the secreted metalloprotease CpaA from *Acinetobacter baumannii*^15^, which contains four tandem repeat modules, and metalloprotease StcE from *E. coli* O157:H7, with a single domain^16^ (Table 2).

**Table 2:** TSP4-N domain homology identified by the program DALI.

Protein	PDB entry code	Z-score	RMSD Cα (Å)	No. aligned Cα pairs	% identity	References
**XD1**						
Phage T4 gp9	1QEX, 1S2E	12.5	2.3	95	18	^12^
Phage T4 gp10	5IV5, 5HX2	5.0	2.8	88	8	^7,13^
*A. baumannii* metalloprotease CpaA	6O38	8.3	2.7	82	20	^15^
*E. coli* O157:H7 metalloprotease StcE	4DNY	6.4	3.0	82	11	^16^
**XD2**						
Phage CBA120 XD3	7REJ	8.7	1.5	60	22	This work
Phage T4 gp10 XD2	5IV5	5.9	1.9	60	10	^7^
Phage T4 gp10 XD3	5IV5	3.4	2.6	59	9	^7^

Both XD2 and XD3 domains of TSP4 adopt β jellyroll folds, which positions the N- and C- β-strands antiparallel and adjacent to one another (Figs. 2A and 3B). The XD2 and XD3 domains share the same threefold symmetry axis as the AD and XD1 domains such that three XD2 domains splay apart and do not interact with one another whereas the three XD3 domains pack together around the threefold symmetry axis. Superposition of the TSP4 XD2 and XD3 domains using the DALI pairwise comparison program resulted in a RMSD of 1.5 Å over 60 aligned Cα atom pairs and 22% amino acid sequence identity (Fig. 3B). The structural and sequence homologies between these two domains exceed any homology to proteins in the PDB identified by the DALI program. Nonetheless, these two domains exhibit structure homology to the XD2 and XD3 domains of phage T4 baseplate gp10 (PDB accession number 5IV5), which is physiologically and evolutionarily relevant to TSP4-N function because in both proteins these domains engage in protein–protein interactions. The DALI pairwise comparison shows that TSP4 XD2 exhibits closer structural similarity to either XD2 and XD3 of gp10 than the TSP4 XD3. The respective XD2 domains of TSP4 and gp10 align with a Z score of 5.9, RMSD of 1.9 Å, and 10% sequence identity over 60 aligned Cα atom pairs. The TSP4 XD2 alignment with gp10 XD3 yields a Z score of 3.4, RMSD of 2.6 Å, and 9% sequence identity over 59 aligned Cα atom pairs. T4 gp10 also functions as a trimer and its XD2 and XD3 domains mediate trimeric protein–protein interactions with T4 baseplate gp12 and gp11 trimers, respectively^7^.

**Figure 4:**
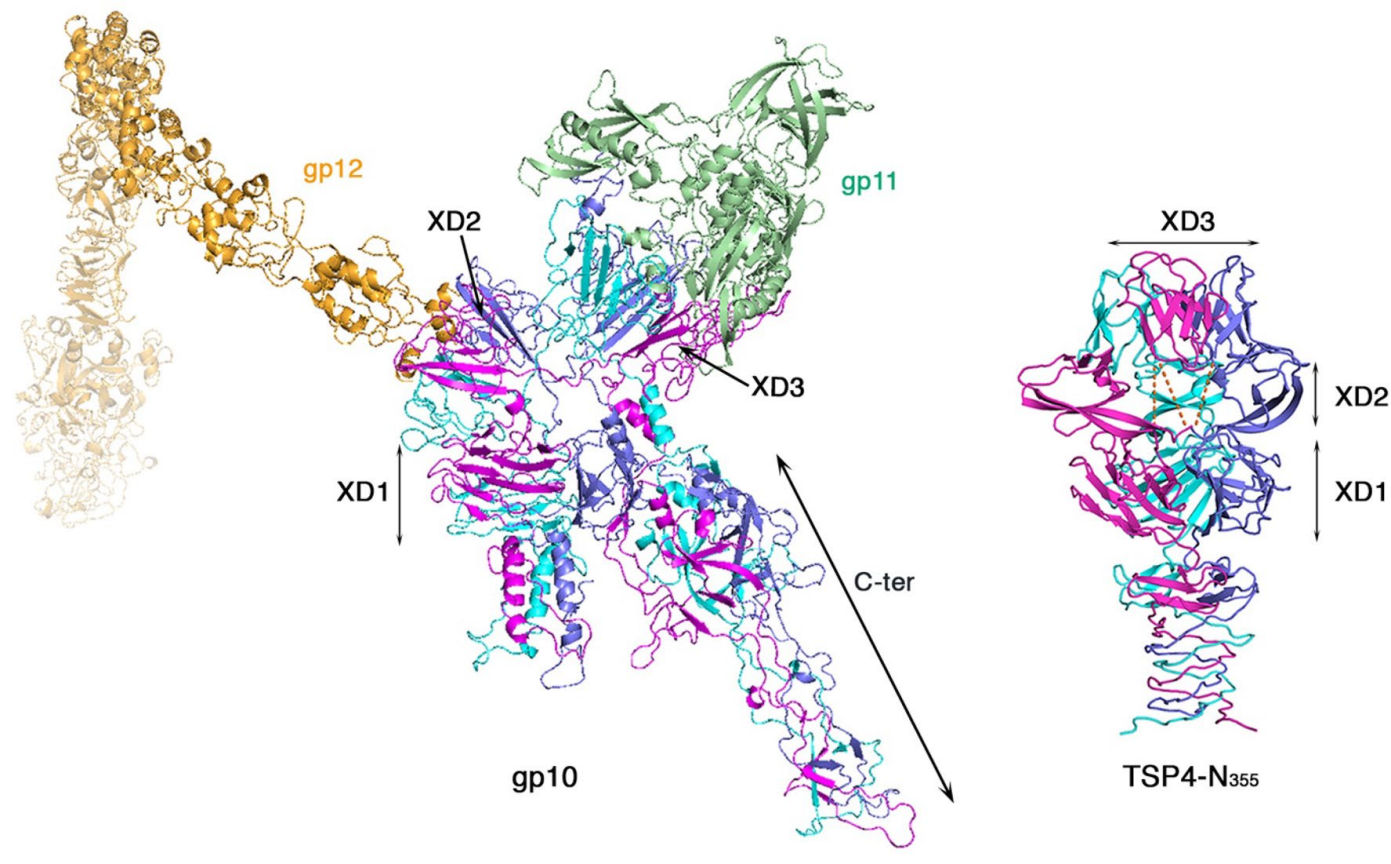
Overall structural similarity between TSP4-N_335_ (right) and phage T4 gp10 (PDB 5IV5) (left) with each subunit shown in different color. The gp10 C-terminal region would have a counterpart in TSP4 D1-D4 region if TSP4 adopted a similar overall shape. All TSP4-N_335_ domains adhere to the same threefold symmetry axis. In contrast, each XD module of gp10 utilizes a different threefold symmetry axis to form differently oriented closely packed trimers. The tail baseplate gp10 partner proteins gp11 (green) and gp12 (orange) bind across the threefold axes of the trimeric XD3 and XD2 domains of gp10, respectively. The XD2 modules of TSP4-N_335_ do not pack together and lack such binding surface. However, the TSP4-N_250_ structure, devoid of the XD3 domain, exhibits closely associated XD2 trimers with a surface that can bind a trimeric partner (Fig. 2C).

The domain orientations in the TSP4-N_335_ and gp10 structures differ because of the utilization of the threefold symmetry axes (Fig. 4). Knowledge of the gp10 3-dimensional domain architecture is crucial for understanding how the TSP complex may assemble as all four domains in the crystal structures of TSP4-N_335_ share the same threefold symmetry axis and the three XD2 modules do not interact with one another (Fig. 2B). The separated XD2 modules lack continuous surface for binding a trimeric partner TSP, which is unlikely to represent the physiological structure. In contrast, the three TSP4 -N_335_ XD3 modules pack closely, with the adjacent N- and C-termini placed within a face perpendicular to the threefold symmetry axis. Consequently, the opposing face provides an uninterrupted trimeric surface where a partner TSP trimer can bind (Figs. 2B and 4). The cryoEM structure of gp10 reveals that the XD2 and XD3 domains obey two different threefold symmetry axes, which is possible if the inter-domain linkers do not comply to exact threefold symmetry (Fig. 4). Each domain exhibits closely packed trimeric modules, which offers two unique uninterrupted surfaces for trimer-trimer interactions with the two gp10 partner proteins, gp11 and gp12. Indeed, the crystal structure of TSP4-N_250_, lacking the XD3 domain, shows three XD2 domains packed together, consistent with the arrangement necessary for promoting trimeric protein–protein interaction (Fig. 2C). Although the intra-domain cores of the XD2 and XD3 modules are tightly packed with primarily hydrophobic amino acids, the inter-domain trimeric interfaces are loosely packed, which suggests that they do not contribute much to the overall trimer stability. The domain separation seen in XD2 of TSP4-N_335_ crystal structures supports this hypothesis.

Taken together, we envision that upon phage CB120 TSP complex formation TSP4-N adopts a domain architecture analogous to that of the gp10 structure as seen in context of the phage T4 tail baseplate (Fig. 4), whereby XD2 and XD3 obey different threefold symmetry axes. Unlike XD2 and XD3, the XD1 modules cannot mediate trimeric protein–protein interactions because their N- and C-termini are located on the opposing faces of the trimer. Consequently, the linkers to the flanking AD and XD2 domains would interfere with binding to a trimeric partner protein across the threefold symmetry axis. Indeed, the XD1 domains of either gp10 or gp9 of phage T4 do not associate with trimeric partners through shared threefold symmetry axes.

Notably, the genome of *E. coli* vB_EcoP_G7C *podoviridae* encodes two TSPs, gp66 and gp63.1 that interact to form a stable, two-branched host recognition complex^17^. Gp66 contains two domains that are expected to adopt the same β jellyroll fold as those of TSP4 as they exhibit 38% and 37% amino acid sequence identities with the TSP4 XD3 domain. Gp66 domain deletion mutants showed that gp63.1 binding to gp66 required the presence of gp66 XD3 domain but not the XD2 domain^17^. Having only gp63.1 as a partner, the role of gp66 XD2 domain remains unknown. For the four CBA120 TSPs, both TSP4 XD2 and XD3 domains are expected to engage in protein–protein interactions.

Four consecutive glycine residues (Gly_76_-Gly_79_) link the TSP4 AD and XD1 domains. The flexibility of this tetra glycine peptide is manifested by the higher temperature factors compared with those of the flanking regions and the slightly different conformations in different crystal forms. This linker along with two other flexible inter-domain linkers described below may assist with accommodating the partner TSP1-3 and with conformational adjustments necessary for optimal cleavage of specific bacterial LPS during phage infection.

A linker (Gly_179_-Leu-Gly-Gln-Gly-Arg-Val-Tyr-Ser-Arg_188_) that connects the XD1 and XD2 domains exhibits a well-defined conformation in the two TSP4-N_335_ crystal structures. The linker extends the N-terminus of the first XD2 β-strand and this extension forms an antiparallel β-β interaction with an extension of the XD2 C-terminal β-strand (Fig. 2A,B). In contrast, the three linkers are conformationally disordered in the crystal structure of TSP4-N_250_, and bring the three XD2 domains together to interact along the crystallographic threefold symmetry axis (Fig. 2C). Presumably, the XD1-XD2 domain orientations may also change in response to changing environment.

The third linker connects the TSP4 XD2 and XD3 domains by a glycine-rich flexible long polypeptide (Thr_250_-Pro-Ile-Gln-Leu-Gly-Asn-Gly-Gly-Gly-Ser-Gly-Ser-Ser-Thr_264_) that is structurally disordered as manifested by the lack of associated interpretable electron density maps for both the wild-type and SeMet TSP4-N_335_ crystal structures.

Vacuum electrostatic calculation using PyMol (The PyMol Molecular Graphics System, Version 1.2r3pre, Schrödinger, LLC) shows that the postulated protein–protein binding surface of the XD3 trimer is negatively charged (Fig. 2E). In contrast, the analogous binding surface of the closely associated XD2 trimer as seen in the TSP4-N_250_ structure, is more neutral, with three lysine residues forming a positively charged patch in the center of the solvent exposed surface (Fig. 2E). As discussed above, the physiologically relevant domain association should comprise closely packed XD2 domains rather than the separated domain organization seen in the TSP4-N_355_ structure, an arrangement that does not allow interaction with a trimeric TSP partner. By analogy to the cryoEM structure of phage T4 gp10, this requires two independent threefold symmetry axes for the XD2 and XD3 domains and the breaking of the threefold symmetry of the linkers between XD1-XD2, XD2-XD3 and XD3-D1 (Fig. 4). Such domain organization generates solvated faces of the trimeric XD2 and XD3 domains, which are available for binding trimeric proteins (Fig. 4A). The different electrostatic properties of these surfaces may facilitate the selectivity for different protein partners. As noted previously, based on the calculated pI values of TSP1-4^2^, the negatively charged binding surface of the TSP4 XD3 trimer complements the N-terminus positively charged surface of the TSP1 head.

### Oligomeric structures of TSP4-N in solution

**Figure 5:**
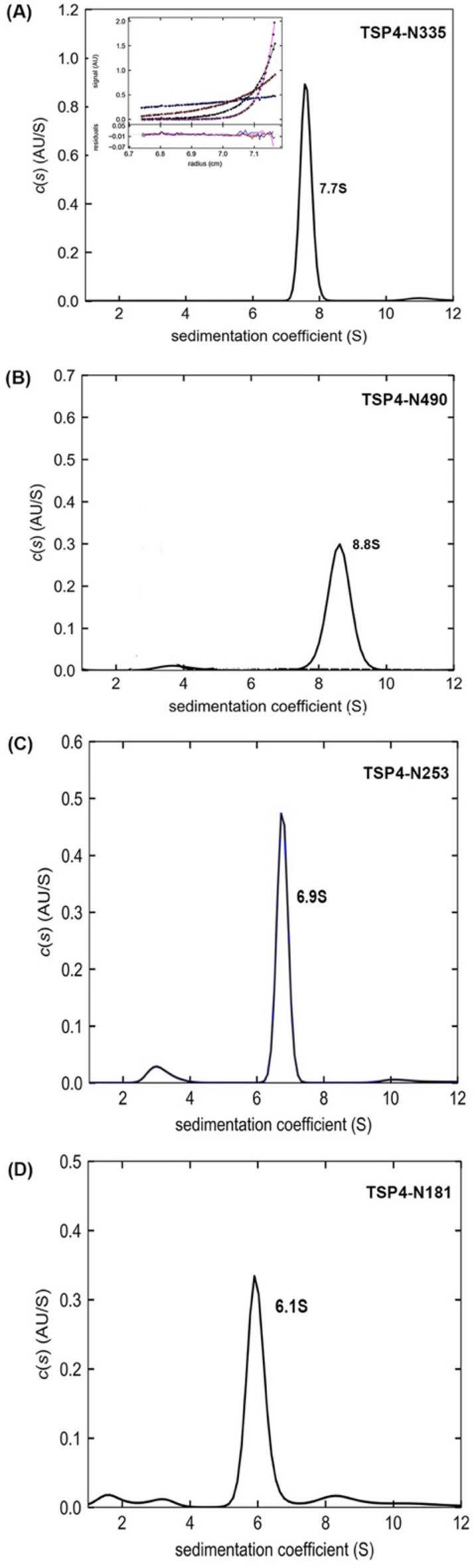
Oligomeric states of TSP4-N proteins. (**A**) The c(s) distribution profile of 3.6 μM oligomeric TSP4-N_335_ showing a single 7.7 S hexamer (dimer of trimers) peak. The insert shows the SE profile of 6.3 μM TSP4-N_335_ oligomer with a best fit RMSD of 0.008 absorbance units (AU), collected at 3, 6, 9 and 12 krpm rotor speeds. All SE profiles were globally analyzed using a single species of interaction system with mass conservation. The best fits are shown as black solid lines through the experimental data. The combined residuals in AU from the same cell at different rotor speeds are shown below the plot. (**B**) The c(s) distribution profiles of 4 μM TSP4-N490. (**C**) 6.8 μM TSP4-N_253_. (**D**) 8.8 μM TSP4-N181.

SDS-PAGE coupled with Western blotting showed that TSP4-N_335_ fractions eluted at high imidazole concentrations contained oligomers, which suggests formation of a stable homomeric complex that remains at least partially folded despite treatment with SDS (Fig. S1A,B). SEC also indicated the presence of monomers and oligomers (Fig. S1C). Subsequently, AUC was used to determine the oligomeric states of TSP4-N recombinant proteins. Sedimentation velocity (SV) experiments showed that the SEC ~ 63 kDa TSP4-N_335_ species had an experimental weight-average sedimentation coefficient, S_*20,w*_, of 2.7 S with the frictional ratio (*f/f*_*0*_) of 1.46 and MW_app_ of 38.7 kDa (Fig. S2A, Table 3). Thus, the TSP4-N_335_ monomer is an elongated molecule. The SEC ~ 470 kDa TSP4-N_335_ species sedimented as a single symmetric peak with S_*20,w*_ of 7.7 S (MW_app_ ~ 199 kDa) (Fig. 5A, Table 3). This result indicated dimerization of TSP4-N_335_ trimers as the MW_calc_ is 218 kDa. Again, the high frictional ratio of 1.7 suggests a highly elongated shape. The complementary sedimentation equilibrium (SE) profile of the TSP4-N_335_ oligomers was best fitted by a single species of interacting system model with MW of 206.5 kDa (Fig. 5A insert). Truncation of the 16N-terminal amino acids (TSP4-N_17-335_) yielded only monomers (data not shown), underscoring the critical role that these residues play in the formation of the triple-stranded ß-helix, a key structural element for TSP4 trimerization. Greater than 50% of the monomeric TSP4-N_335_ sample converted into oligomers with S_*20,w*_ values of 5.2 S and 7.7 S when stored at high concentration and 4 °C for a few weeks (Fig. S2A). It is also interesting to note that circular dichroism (CD) analysis of the fraction containing protein monomers showed a signature β-sheet profile characterized by a minimum at 217 nm (Fig. S2B), suggesting that the XD domains are folded. The region that ultimately forms the intertwined triple-stranded β-helix may fold slowly and govern the rate of oligomerization. The elongated TSP4-N_335_ hexamer determined by x-ray crystallography agrees with the elongated shape derived from the SV results (Table 3).

**Table 3:** SV results for phage CBA120 TSPs.

Protein	Oligomeric form	MW_calc_ (kDa)^a^	S_20,w_ (S)^b^	MW_aap_ (kDa)^c^	*f/f*_*0*_^b^
TSP1	Trimer	248	9.6	276	1.55
TSP1_14-166_	Trimer	50	3.6	53	1.43
TSP2	Trimer	297	9.9	279	1.56
TSP3	Trimer	206	8.4	195	1.43
	Hexamer	412	12.9	357	
TSP3_156-627_	Trimer	180	7.7	140	1.25
	Hexamer	360	12.9	300	
	Nanomer	540	17.7	490	
TSP4-N_335_	Monomer	36	2.7	39	1.46^d^
	Trimer	109	5.2	86	1.33^d^
	Hexamer	218	7.7	199	1.67^d^
TSP4-N_490_	Monomer	53	3.1	39	1.58
	Hexamer	316	8.8	264	1.69
TSP4-N_181_	Hexamer	120	6.1	109	1.45
TSP4-N_253_	Hexamer	167	6.9	125	1.36
TSP4_185-1036_	Trimer	273	9.8	201	1.27

^a^Molar mass calculated from the amino acid composition of the proteins.

^b^S_20,w_ and best fitted frictional ratio (*f/f*_*0*_) values from the SEDFIT analyses using the continuous *c(s)* distribution model.

^c^Experimental molar mass distribution *c(M)* values from the SEDFIT analyses using the continuous *c(s)* distribution model. Note that the *c(M)* values are correlated to the *f/fo* values, which are subjected to the number and shapes of species present in the sample.

^d^TSP4-N_335_ monomers and hexamers are purified as single species. Trimeric TSP4-N_335_ exists in the presence of monomeric and hexameric species.

Consistent with the solution properties, the crystal packings of both the TSP4-N_335_ and the TSP4-N_250_ trimers stack the flat surfaces of the two AD termini face-to-face. The twofold symmetry axis of this dimer of trimers runs perpendicular to the trimer threefold symmetry axis (Fig. 2D). The dimer interface contains primarily hydrophobic amino acid residues (Fig. 2E, bottom). Three phenylalanine residues at the core, as well as six prolines and six leucine residues at the edge, line the N-terminal triple-stranded β-helix layer of each trimer. Interestingly, the negative-stained EM images showed that full-length TSP4 also forms similar dimer of trimers when complexed with TSP2^2^. In contrast, the staining-EM images of the TSP1-4 complex as well as images of the intact phage CBA120 show that the complex comprising all four TSPs has no two-fold symmetry axis. It is tempting to speculate that the hydrophobic N-terminal surface anchors the TSP complex to the tail baseplate.

TSP4-N_490_ contains the four TSP4-N_335_ domains followed by the D1-D2 head region (Fig. 1). As TSP4-N_335_, TSP4-N_490_ is an elongated stable hexamer (Fig. 5B, Table 3). However, the monomers were susceptible to proteolytic degradation, preventing analysis of their transition into higher oligomeric forms.

Two other TSP4-N fragments were prepared to identify binding specificities of the XD2 and XD3 domains towards TSP1-3. TSP4-N_253_ comprises AD-XD1-XD2, and TSP4-N_181_ comprises AD-XD1 (Fig. 1). For both, the experimental sedimentation parameters agree with the calculated parameters base on the crystal structure (Fig. 5C,D, Table 3).

### Interpretation of the AUC binding data

The experimental S_*20,w*_ and molecular weights (MW_app_) of individual TSPs derived from SV data are consistent with the calculated S_*20,w*_ and molecular weights (MW_calc_) (Table 3). However, mixtures of proteins of various binding affinities contain heterogenous combinations of complexes and free proteins with experimental MW_app_ values that are lower than the MW_calc_ values (Table 4), which impedes the stoichiometry determinations. This is not surprising because the MW_app_ values are derived from a single weight-average *f/f*_*0*_. Moreover, the experimental S_*20,w*_ values of weak and transient protein complexes reflect the coupled migration of dynamically exchanging free and bound complexes, in contrast to high affinity complexes that sediment rapidly^18^. Therefore, an increase in the averaged S-values in the protein mixture as a function of protein concentration indicates the formation of complexes, but not their true S-values or sizes. With the available AUC instrumentation and protein supply limitations, the approach taken in this study was to establish the S_*20,w*_ values of single TSPs and then assign the S_*20,w*_ of new peaks by gradually examining binary, ternary and quaternary complexes. For domain-deleted TSPs used to identify the specificity of domain-domain interactions, absence of new peaks confirms elimination of interactions. The *c*(s) profiles are shown in the figures and peak assignments are summarized in Tables 3 and 4. Detailed results and rationale for peak assignments are provided in the Supplementary Information.

**Table 4:** SV results for CBA120 TSP complexes.

Protein mixture	Stoichiometry	MW_calc_ (kDa)^a^	S_20,w_ (S)^b^	MW_app_ (kDa)
TSP1:TSP4-N_335_	3:6	466	11.4	431
	6:6	714	14.3	615
TSP1:TSP4-N_490_	3:6	564	12.4	484
	6:6	812	15.7	686
TSP1_14-166_:TSP4-N_335_	3:6	268	8.4	139
	6:6	318	11.3	220
TSP2:TSP4-N_335_	3:6	515	12.9	392
	6:6	802	15.0	527
TSP2:TSP4-N_490_	3:6	613	14.3	453
	6:6	910	16.7	571
TSP2:TSP4-N_253_	3:6	464	12.1	340
	6:6	761	13.6	407
TSP2:TSP4-N_181_	3:6	417	12.0	292
	6:6	714	13.8	413
TSP1:TSP2:TSP4-N_335_	6:3:6	1011	17.4	708
	6:6:6	1308	19.0	870
TSP2:TSP3:TSP4-N_335_	3:3:6	725	15.6	603
	6:6:6	1224	18.8	831
TSP1:TSP2:TSP3:TSP4-N_335_	6:6:3:6	1514	19.7	634
	6:6:6:6	1720	23.8	847

^a^Molar mass calculated from the amino acid composition of the proteins.

^b^S_20,w_ values from the SEDFIT analyses using the continuous *c(s)* distribution model.

### TSP4-N binary complexes

**Figure 6:**
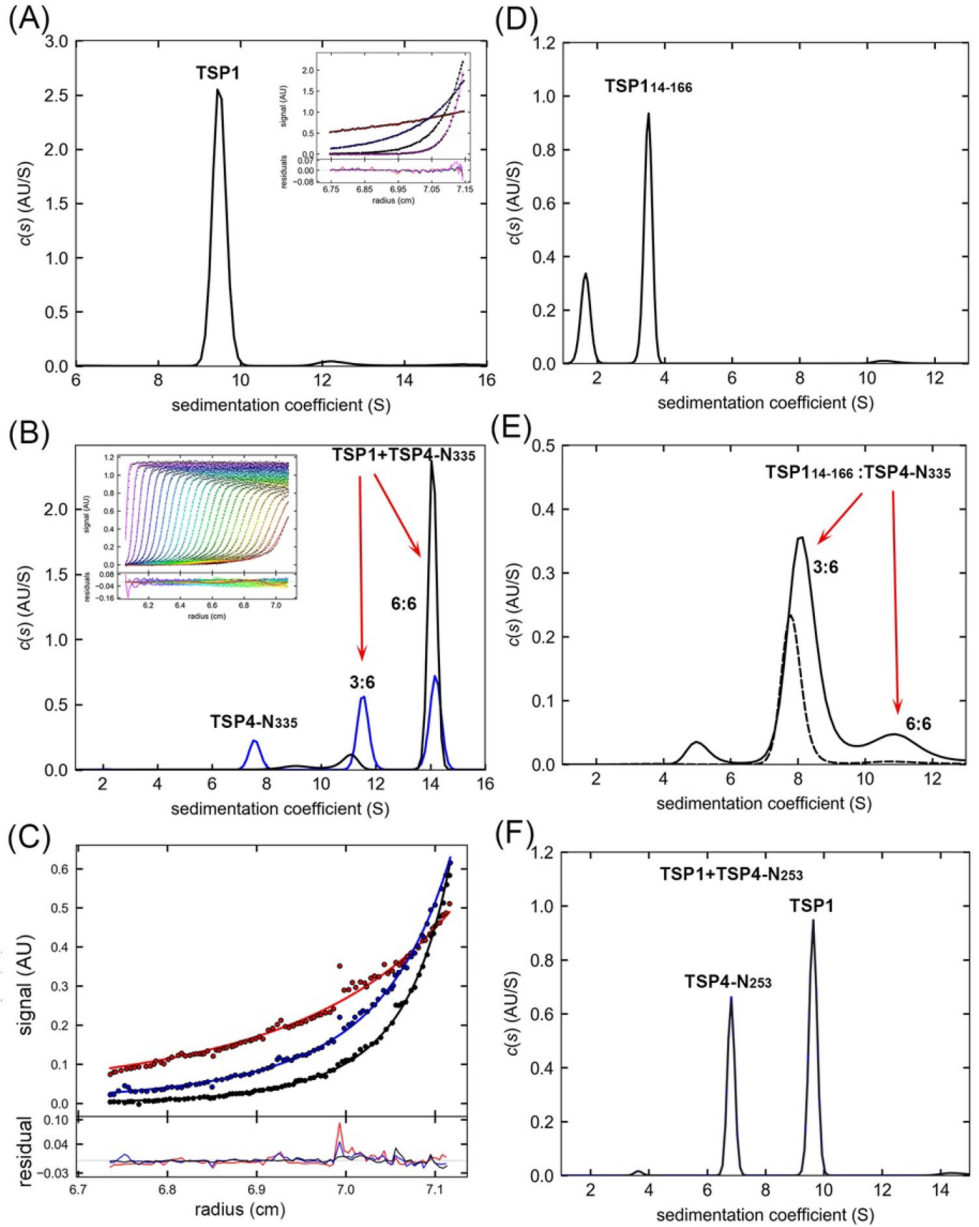
AUC analyses of TSP1 and its association with TSP4-N proteins. **(A)** The *c*(*s*) distribution of 8.1 μM TSP1. Insert: SE profile of 3 μM TSP1 with a best fit RMSD of 0.012 AU, collected at 3, 6, 8 and 12 krpm rotor speeds. The best fits are shown as black solid lines through the experimental data with MW_app_ of 226 kDa. **(B)** The *c*(s) distribution profiles of 3.6 μM TSP4-N_335_ hexamer with 2.8 μM TSP1 (blue) and with 6.4 μM TSP1 (black). Insert: Direct Lamm-Equation modeling of the SV experiment containing 3.6 μM TSP4-N_335_ and 6.4 μM TSP1. The best fits are shown as solid lines through the experimental data. **(C)** SE profile of 1.0 μM each of TSP4-N_335_ and TSP1 with a best fit RMSD of 0.011 AU, collected at 4, 6 and 8 krpm rotor speeds. The best fits are shown as solid lines through the experimental data with K_d_ = 0.08 μM. The LEq analysis of SV data and that of SE profiles were calculated with global best-fit distributions using a [A + B + B ↔ AB + B ↔ ABB] model with 2 symmetric sites and macroscopic association constant *K*_A_. The combined residuals in AU from the same cell at different rotor speeds are shown below the plot. **(D)** The *c*(*s*) distribution profiles of 3.3 μM TSP1_14-166_, missing the N-terminal 14 amino acids, in the presence of 100 μM ZnCl_2_. **(E)** The *c*(*s*) distribution profiles of a mixture of 3.3 μM TSP4-N_335_ (dashed line) and of a mixture of 3.3 μM TSP4-N_335_ and 13 μM TSP1-N_14-166_ (solid line). (**F**) The *c*(*s*) distribution profiles of a mixture of 5.4 µM TSP4-N_253_ and 3.3 µM TSP1.

The SV experiments showed that TSP1 homotrimers sedimented as a 9.6 S species (Fig. 6A, Table 3)^4^. The SV analysis of a TSP1 and TSP4-N_335_ mixture showed two peaks with S_*20,w*_ of 11.4 S and 14.3 S that correspond to binding of one and two TSP1 trimers to the TSP4-N_335_ hexamer, respectively (Fig. 6B, Table 4). The Lamm Equation (LEq) analysis of the SV data was best fitted with *K*_d_ = 0.06 ± 0.06 μM, *k*_off_ = 10^–4^ s^−1^ using a two-site heterogeneous association model [A + B + B ⇆ AB + B ⇆ ABB], where A and B correspond to TSP4-N_335_ hexamer and TSP1 trimer, respectively (Fig. 6B insert). The global fitting of SE experiments gave *K*_d_ = 0.08 μM using this model (Fig. 6C).

The TSP4-N_253_ fragment containing the AD-XD1-XD2 domains but lacking the XD3 domain sedimented at S_*20,w*_ of 6.9 S (Fig. 5C, Table 3). The SV profile of a TSP1 and TSP4-N_253_ mixture shows peaks that correspond only to free proteins (Figs. 5C, 6A,F). The absence of complex peaks indicates that the binding site for TSP1 resides on the XD3 domain.

Because the calculated pI of the TSP4 XD3 domain is much lower than that of the XD2 domain, Plattner and colleagues hypothesized that the positively charged TSP1 head interacts with the TSP4 XD3 domain^2^. The TSP4-N_335_ crystal structure reveals that indeed, the charge distribution of the respective surfaces complements one another (Fig. 2E of this work, and^4^). To test this hypothesis, we prepared two versions of the TSP1 head, TSP1-N_166_ and TSP1-N_14-166_, comprising the D1-D2 domains and the ensuing α-helical neck region with and without the 13N-terminal amino acids, which are disordered in the TSP1 crystal structure (Fig. 1)^4^. The addition of Zn^2+^, which is bound to the D1 N-terminal α-helix in the crystal structure (PDB accession number 4OJ5), promoted the trimerization of the TSP1 head region (Fig. 6D). The SV analysis of the TSP1-N_14-166_ and TSP4-N_335_ mixture in the presence of Zn^2+^ confirmed TSP1-N_14-166_:TSP4-N_335_ complex formation (Fig. 6E). The TSP1-N_166_ and TSP4-N_335_ mixture precipitated, thus the contribution of the TSP1 13N-terminal residues to TSP4-N_335_ binding remains unknown.

**Figure 7:**
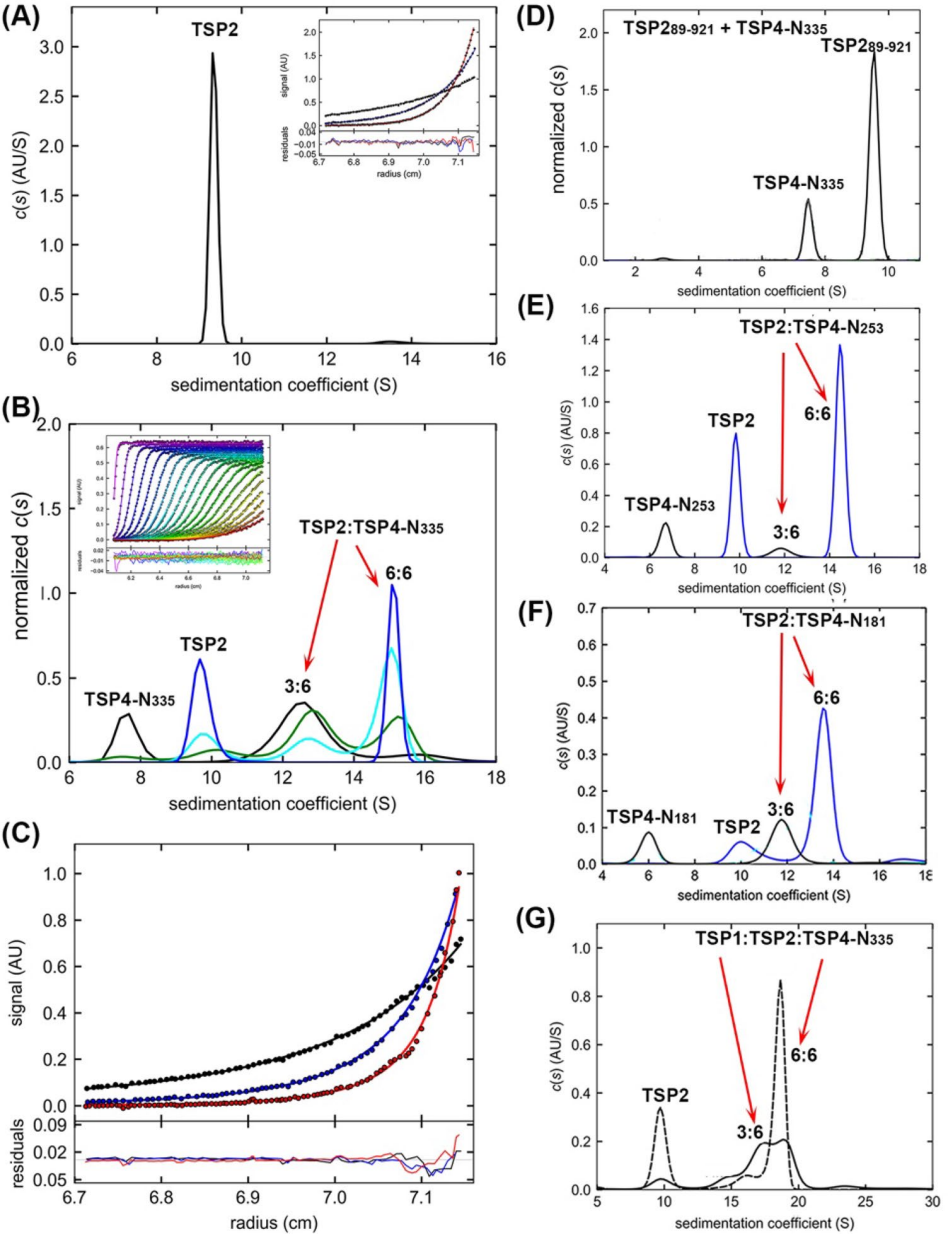
AUC analyses of the association between TSP2 and TSP4-N proteins in the absence and presence of TSP1. (**A**) The *c*(*s*) distribution of 3.5 μM TSP2. Insert: SE profile of 3 μM TSP2 with a best fit RMSD of 0.01 AU, collected at 4, 6 and 8 krpm rotor speeds. The best fits are shown as black solid lines through the experimental data with MW_app_ of 270 kDa. (**B**) The SV profiles of 3.3 μM TSP4-N_335_ in the presence of different TSP2 concentrations: 1.7 μM (black), 3.3 μM (green), 5 μM (cyan), and 8 μM TSP2 (blue). Insert: Direct LEq modeling of SV data from 3.3 μM each of TSP2 and TSP4-N_335_ hexamer. The best fits are shown as solid lines through the experimental data. (**C**) SE profile of 1.3 μM TSP4-N_355_ and 1.3 μM TSP2 with a best fit RMSD of 0.012 AU, collected at 4, 6 and 8 krpm rotor speeds. The best fits are shown as solid lines through the experimental data. The LEq analysis of SV and SE data calculated with the program SEDPHAT are described in Fig. 6C. (**D**) The *c*(*s*) distribution profiles of 3 μM TSP4-N_335_ with 2 μM TSP2_89-921_ lacking the XD2 domain. (**E**) The *c*(*s*) distribution profiles of 3 μM TSP4-N_253_ in the presence of 0.37 μM (black) and 2.5 μM TSP2 (blue). (**F**) The SV profiles of 3.16 μM TSP4-N_181_ in the presence of 0.37 μM (black) and 1.5 μM TSP2 (blue). (**G**) The *c*(*s*) distribution profiles of 3.3 μM each of TSP4-N_335_, TSP1 and TSP2 (solid line), and 3.3 μM each of TSP4-N_335_ and TSP1 with 6.6 μM TSP2 (dashed line).

TSP2 sedimented as homotrimers with S_*20,w*_ of 9.9 S (Fig. 7A, Table 3). SV experiments of the TSP4-N_335_ hexamer and TSP2 mixtures exhibited two new peaks (Fig. 7B, Table 4), attributed to TSP2:TSP4-N_335_ complexes with 3:6 and 6:6 stoichiometry, respectively. The LEq modeling of the TSP2:TSP4-N_335_ SV data assuming a two-site heterogeneous association model gave *K*_d_ = 0.11 ± 0.8 μM, *k*_off_ = 2.5 × 10^–4^ s^−1^. The global fitting of the SE data gave *K*_d_ = 0.075 μM with the same model (Fig. 7C).

Next, we identified which domain of the 170-residue TSP2 N-terminal region preceding the single D1 head domain (Fig. 1) recognizes the TSP4-N_335_. Both BLAST^19^ and the profile hidden Markov models program HHpred^20^ reveal that the N-terminal TSP2 region comprises two domains homologous to the XD2 and XD3 domains of phage T4 gp10 baseplate protein. Pairwise sequence alignment with the program LALIGN^21^ shows that the TSP2 XD2 domain exhibits 29% sequence identity to the TSP4 XD3 domain over 55 aligned residue, and the TSP2 XD3 domain exhibits 33% sequence identity to the TSP4 XD3 domain over 51 aligned residues. Plattner and colleagues proposed that the association between the TSP2 and TSP4 occurs via their respective XD2 domains^2^. AUC experiments using a TSP2 with a deleted XD2 domain, TSP2_86-921_ probed these interactions (Fig. 1). A TSP4-N_335_ and TSP2_89-921_ mixture showed only the free proteins (Fig. 7D), confirming that the TSP2 XD2 domain mediates the TSP2 binding to TSP4.

TSP2 binds to TSP4-N proteins devoid of the XD3 domain. The SV analyses showed two TSP2:TSP4-N_253_ complexes, and two TSP2:TSP4-N_181_ complexes (Fig. 7E&F). The binding of TSP2 to TSP4-N_181_ is surprising as it lacks the XD2 domain and the AD-XD1 domains have no trimeric surface for protein–protein interaction except for the baseplate anchoring surface that mediates hexamer formation. A protein devoid of the TSP4 AD-XD1 domains (TSP4_185-1036_) did not bind TSP2. Perhaps the weak interactions between the XD2 subunits requires the presence of the AD-XD1 domains, in particular the triple-stranded β-helix, for trimeric assembly. On the other hand, the side face of TSP4 AD-XD1 is enriched with negatively charged residues (Fig. 2E) and may provide a non-specific electrostatic interaction surface on the non-physiological TSP4-N_181_ fragment, which can complement the positively charged residues on the TSP2 XD2 surface.

**Figure 8:**
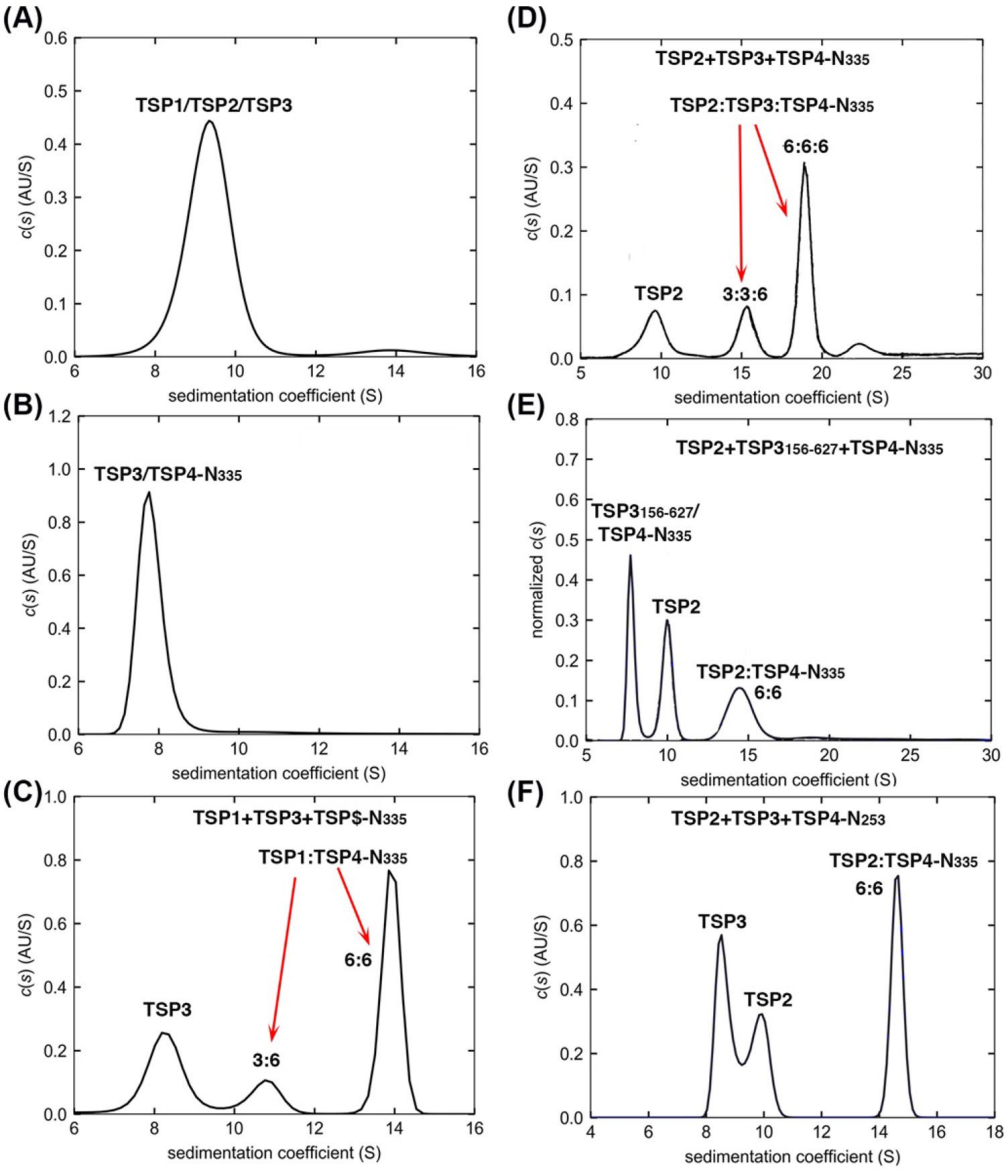
SV analyses of the interactions of TSP3 with partner TSPs (**A**) The *c*(*s*) distribution profile of a mixture containing 1.5 μM each of TSP1, TSP2 and TSP3 shows no interactions between these proteins. (**B**) The *c*(*s*) distribution profile of 5 μM each of TSP4-N_335_ and TSP3 shows no association between the two proteins. (**C**) The *c*(*s*) distribution profile of 5 μM each of TSP4-N_335_, TSP1 and TSP3 revealed no association between TSP3 and the TSP1:TSP4-N_335_ binary complex. (**D**) The SV profile of 1.5 μM each of TSP4-N_335_, TSP2 and TSP3 shows a ternary complex formation. (**E**) The *c*(*s*) distribution profile of 3 μM TSP4-N_335_, 2.5 μM TSP2 and 3 μM TSP3_156-627_ shows no ternary complex when the TSP3 head is absent. (**F**) The SV profile of a mixture containing 3.1 µM TSP4-N_253_, 2.5 µM TSP2 and 2.5 µM TSP3 shows that TSP3 does not bind to the TSP2:TSP4-N_253_ binary complex lacking the TSP4 XD1 domain.

Unlike TSP1 and TSP2, TSP3 does not associate directly with TSP4-N_335_ as shown by the broad peaks that correspond only to the free proteins (Fig. 8B).

### Ternary complexes

The TSP1, TSP2 and TSP4-N_335_ mixture showed two new peaks (Fig. 7G, solid line), which are attributed to the formation of ternary TSP1:TSP2:TSP4-N_335_ complexes at 6:3:6 and 6:6:6 stoichiometry. Again, the D1-D2 head domains of TSP4 did not impede the binding of TSP1 and TSP2 (Fig. S3D).

TSP1, TSP2 and TSP3 do not interact with one another in the absence of TSP4-N (Fig. 8A). Consistent with the negative-staining EM^2^, the AUC studies show that TSP3 binding requires a preformed TSP2:TSP4-N complex. The SV analysis showed two peaks that were attributed to 3:3:6 and 6:6:6 TSP2:TSP3:TSP4-N_335_ complexes (Fig. 8D, Tables 3, 4). In contrast, no ternary complex formed with a mixture that contained TSP1 instead of TSP2 (Fig. 8C).

A truncated TSP3 lacking the head, TSP3_156-627_, was used to demonstrate that TSP3 binding to the TSP2:TSP4-N_335_ complex is mediated by the head region of TSP3 because we were unable to produce a trimeric TSP3 D1-D2 head (Fig. 1). The SV analyses of the TSP2, TSP3_156-627_ and TSP4-N_335_ mixture showed peaks representing free TSP3_156-627_, free TSP2 and a TSP2:TSP4-N_335_ complex (Fig. 8E). The peaks corresponding to ternary complexes (Fig. 8D) were absent, which confirmed that the binding of TSP3 to the TSP4-N_335_:TSP2 complex requires the presence of the TSP3 head.

As TSP2 XD2 domain mediates TSP2 binding to TSP4-N, the adjacent TSP2 XD3 surface is available for binding the TSP3 trimer. However, the SV experiments reveal that TSP3 binding requires the presence of TSP4-N XD3 domain even though this domain already serves as the TSP1 binding site. A mixture of TSP2, TSP3 and TSP4-N_253_ lacking the XD3 domain showed no peak that may be attributed to a ternary complex (Fig. 8F).

### The quaternary TSP complex

**Figure 9:**
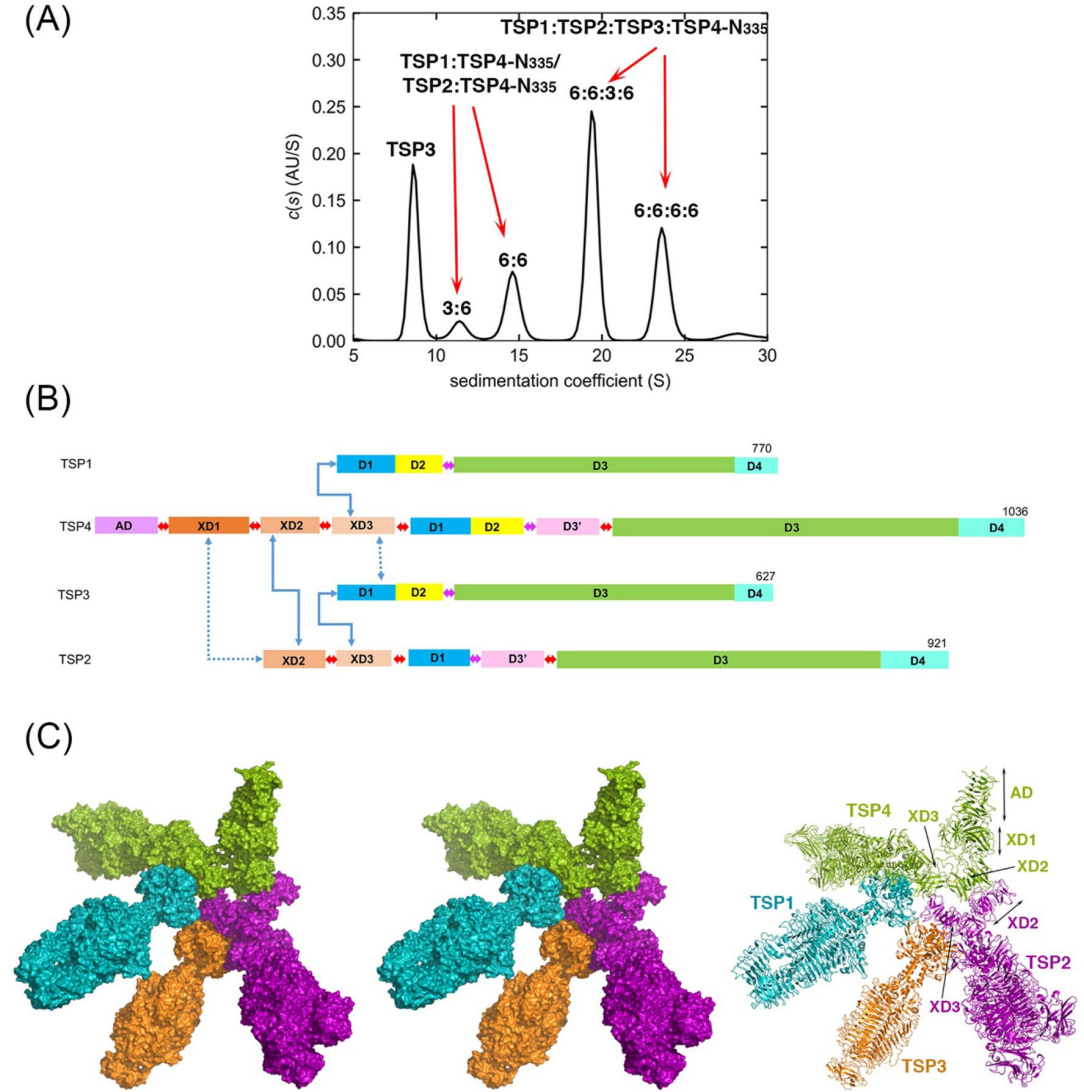
The phage CBA120 TSP quaternary complex. (**A**) The *c*(*s*) distribution profiles of 3.3 μM each of TSP1, TSP2, TSP3 and TSP4-N_335_. (**B**) Summary of the protein–protein interactions revealed by the AUC experiments. TSP domains are colored as in Fig. 1. Primary domain-domain interactions that presumably utilize the same threefold symmetry axes are indicated by solid blue arrows. Protein–protein interactions that presumably do not occur through shared threefold symmetry axes are indicated by dashed blue arrows. (**C**) Molecular model of the quaternary TSP complex. Left: Stereoscopic representation of the space filling model showing TSP1 (blue), TSP2 (magenta), TSP3 (orange) and TSP4 (green). Right: Ribbon representation using the same color scheme.

The *c*(*s*) distribution profiles of a TSP1, TSP2, TSP3 and TSP4-N_335_ mixture showed two new peaks attributed to TSP1:TSP2:TSP3:TSP4-N_335_ complexes (Fig. 9A). This complex, as well as all binary and ternary complexes described above were also formed with TSP4-N_490_, which contains the TSP4 D1-D2 head (Fig. S3). Thus, the presence of TSP4 head does not interfere with partner TSP binding. Figure 9B summarizes schematically these interactions (1) The D1 domain of TSP1 head binds to TSP4 XD3, (2) TSP2 XD2 domain binds to TSP4-N, (3) Although not necessarily physiological, an unknown region of TSP2 interacts with TSP4 AD-XD1, and (4) the TSP3 head binds to TSP2:TSP4-N complex only in the presence of TSP4 XD3.

The AUC results, the crystal structures, along with the negatively-stained EM branched structures and bioinformatic analyses guided the modeling of a three-dimensional complex comprising all four phage CBA120 TSPs (Fig. 9C). Because all domains of the TSP4-N_335_ crystal structure are placed around the same threefold symmetry axis, the trimer’s XD2 domains do not interact with one another and TSP2 binding to a trimeric TSP4 XD2 is precluded. Nevertheless, the TSP4-N_250_ crystal structure shows that the TSP4 XD2 domain can form a closely packed trimer and the flexible linkers can adjust the relative orientations between the globular trimeric domains (Fig. 2C). Therefore, the EM structure of phage T4 gp10 (Fig. 4) and its modes of interactions with gp11 and gp12 provided a template for the TSP quaternary complex model. For the full length TSP2 model, homology models of the XD2 and XD3 domains were built with the TSP4 XD3 domain as a template. The modeling protocol is described in detail in the Supplementary Information.

Notably, the positively charged N-terminus of the D1 domain of the TSP1 head compliments the negatively charged TSP4 XD3 surface (Fig. 2E). The TSP4 XD2 trimer binding surface, as seen in the TSP4-N_250_ crystal structure, exhibits positively charged residues at the center and negatively charged residues at the rim (Fig. 2E). The modeled TSP2 XD2 trimer exhibits the reverse trend; negatively charged residues at the center and positively charged residues at the rim. The AUC analysis shows that the TSP2 XD3 domain does not interact with TSP4-N_335_ (Fig. 7D), and thus it can provide a surface for binding TSP3. In the modeled complex (Fig. 9C), the TSP2 XD3 trimer was placed in between the TSP4 XD3 and TSP2 XD2 trimers to account for the experimental finding that TSP3 binding to TSP4-N requires the presence of TSP4 XD3. Restricting the TSP2 XD3 trimer in this wedged position allows the TSP3 head to form the primary interaction with bound TSP2 and also to interact with TSP1 heads, providing further stability to the quaternary complex.

## Conclusions

The bacteriophage CBA120 multi-TSP complex serves as a paradigm for the host recognition apparatus of other *Kutterviruses* that encode multiple TSPs as well as phages from other families whose genomes encode more than a single tailspike, for example phage G7C. From a broader perspective, the structures of TSP4-N and bacteriophage T4 baseplate proteins highlight how similar protein domains may perform related functions in entirely different contexts. For example, the triple-stranded β-helix of the TSP4 AD domain promotes trimerization as deletion of the 12 N-terminal amino acid residues prevents oligomerization of TSP4-N_355_. In different contexts, triple-stranded β-helices are utilized by a number of fibrous phage proteins, including the gp12 short tail fiber protein, the gp34 proximal long tail fiber protein, and the cell puncturing segment of gp5 of phage T4. Because of the intertwining of the three protomers, this motif may enhance protein trimer stability. The XD2 and XD3 modules also demonstrate the diverse employment of the same folding motif in different contexts. In the phage T4 gp10 baseplate protein, trimeric XD2 and XD3 modules provide the binding sites for the baseplate wedge protein gp11 and the short tail fiber gp12, respectively. In phage CBA120, the TSP4 XD2 and XD3 modules provide the binding sites for TSP2 and TSP1, respectively. It appears that the β-jellyroll module has been selected as a trimer assembly motif for interactions with other phage trimeric proteins. The function of the XD1 module is not yet fully understood. In addition to TSP4 and gp10, a trimeric XD1 is also present in phage T4 gp9, a baseplate protein that associates with the long tail fiber protein. In these cases, the XD1 modules appear to serve as spacers between modules that engage in protein–protein interactions.

We hypothesize that the flexible inter-domain linkers of TSP4-N play a key role in changing the orientations of the XD2 and XD3 trimers. This inherent linker flexibility is critical to the function of *Kuttervirus* TSP branched complexes because these phages infect multiple bacterial strains that are coated by different LPSs, and the TSP assembly at the tail end needs to adjust in response to different environments in order to cleave different polysaccharides.

The AUC analyses identified TSP interactions that mostly agree with the assembly model proposed by Leiman and colleagues^2^, with the exceptions that TSP1 binds directly to TSP4 and does not require a preformed TSP2:TSP4 complex. TSP1 and TSP3 bind to TSP4 and TSP2:TSP4, respectively, via their head domains, whereas TSP2 binds to TSP4 via its N-terminal XD2 domain. Binding of TSP1 is mediated by the TSP4 XD3 domain. In addition to binding to TSP4 AD-XD1-XD2, TSP2 also binds to TSP4 AD-XD. The TSP2 interaction with the AD-XD fragment is puzzling. Structure of the TSP1-4 complex will reveal whether this interaction occurs when all domains are present.

Finally, phages encoding multivalent TSPs may be exploited for therapeutic and industrial purposes by engineering different multi-host specificities. For example, a replacement of one of the TSPs with another TSP that enables the phage to grow on a common laboratory bacterial strain would provide a useful tool for scaling up phage production. With the current studies, we have laid down the foundation for further development that will hopefully accomplish this goal.

## Materials and methods

### Cloning, production, and protein purification

The nucleic acid sequences of TSP4-N_335_, TSP4-N_490_ and a TSP4-N_335_ L12M:I31M:L145M mutant gene with three additional methionine residues were codon-optimized for expression in *E. coli*, and synthesized by GeneArt (ThermoFisher). These genes and domain-deleted TSP4-N constructs were sub-cloned into a pBAD24 expression vector and recombinant proteins with C-terminal 6x-His tags were produced in BL21*(DE3) or Rosetta-gami 2 cells. Proteins were produced and purified using Ni-affinity chromatography followed by SEC as previously published^4–6^.

### Analytical SEC

TSP4-N_335_ analytical SEC was performed with a Superdex 200 HR 10/30 column (GE Healthcare). The elution coefficient, *K*_av_, is defined as (V_e_ − V_o_)/(V_t_ − V_o_), where V_e_ is the elution volume for the protein, V_o_ is the excluded void volume and V_t_ is the total volume of the column. The standard curve was calculated by plotting the apparent molecular weight of standard proteins (MW_app_) as a function of their elution coefficients, *K*_av_, and used to estimate the MW_app_ of N-terminal domains of TSP4 from their elution coefficients.

### Analytical ultracentrifugation (AUC)

SV and SE experiments were performed using a ProteomeLab Beckman XL-A with absorbance optical system and a 4-hole An60-Ti rotor (Beckman Coulter). For SV, 380 μL protein in PBS, pH 7.4, and 400 μL buffer were loaded into the sample and reference sectors of the dual-sector charcoal-filled epon centerpieces. The samples were equilibrated overnight at 20 °C. For the effects of Zn^2+^ ions, the SV analyses were conducted in 50 mM TRIS (pH 8.0) or HEPES (pH 7.3) buffer containing 150 mM NaCl and 0.1–0.5 mM Zn^2+^. The samples were centrifuged at 30–50 krpm and the absorbance data for 0.125–30 μM proteins were collected at 280 nm to obtain linear signals of < 1.25 absorbance units. Absorbance signal was monitored in a continuous mode with a step size of 0.003 cm and a single reading per step. Sedimentation coefficients were calculated from SV profiles using the program SEDFIT^22^. The continuous *c*(*s*) distributions were calculated assuming a direct sedimentation boundary model with maximum entry regularization at a confidence level of 1 standard deviation. The LEq analyses of SV data were conducted using the Hybrid Local Continuous and Global Discrete Species model for molecules A or B alone to obtain molecular mass and sedimentation coefficients^23^. These values were subsequently used in the hetero-association analysis with a two-site heterogeneous association model [A + B + B ⇆ AB + B ⇆ ABB] to obtain the equilibrium dissociation constant, *K*_d_, off rate constant for the complexes, *k*_*off*_ and the sedimentation coefficient of complexes *s*AB *and s*ABB, where A is a TSP4-N_335_ hexamer and B is a partner TSP trimer with equilibrium association constants for AB and ABB complexes of *K*_A_(1) and *K*_A_(2), respectively^23^. The model assumes no cooperativity between the two sites and the off rates for AB and ABB complexes are the same.

For SE, the sample and reference sectors of dual-sector centerpieces were filled with 170 μL protein (0.5–14 μM) and with 180 μL PBS, respectively. Each SE experiment was conducted at 3 or 4 speeds (3000–12,000 rpm) at 20 °C, increasing from the lowest to the highest speed. Equilibrium was considered as reached when the RMSD value of successive scans taken at 3-h periods was below the noise level as determined by SEDFIT. Absorbance was scanned at wavelength intervals of 0.001 cm with 20 replicates per step. The SE curves were analyzed using the non-linear regression analysis program SEDPHAT to obtain the *K*_d_, based on the Boltzmann distributions of ideal species in the centrifugal field^24^.

The integrity of protein samples before and after the AUC experiments were assessed using SDS-PAGE and Western blot assays under non-denaturing and denaturing conditions. The density and viscosity of buffers at 20ºC and 4ºC were calculated using SEDNTERP^25^. The structure-based hydrodynamic properties of proteins were calculated using the bead shell-modeling program HYDROPRO^26^. The *c*(*s*) distributions and SE profiles were prepared with the program GUSSI^27^.

### Crystallization, data collection, and structure determination

Crystals of TSP4-N_335_ and TSP4-N_250_ were obtained at 22 ºC using the vapor diffusion method with 6–10 mg/mL protein concentrations. The drops contained equal volumes of protein solution and mother liquor. Both wild-type and SeMet TSP4-N_335_ formed diffraction quality crystals in two different conditions: (1) 1.0 M KNa tartrate, 0.2 M NaCl, 0.1 M imidazole (pH 7.8) (space group R 3 (146)), and (2) 1.6 M Li_2_SO_4_, 0.1 M NaCl 0.1 M HEPES (pH 7.5), (space group P 4_1_ 2_1_ 2). Crystals of TSP4-N_250_ were obtained in solutions that initially contained TSP4-N_490_, which degraded during crystal growth, with 20% w/v polyethylene glycol 8000, 0.2 M NaCl, 0.1 M CHAPS (pH 10.5) (space group R32 (155)).

For data collection, crystals were transferred to mother liquor supplemented with 10–30% (v/v) glycerol and flash-cooled in liquid nitrogen. Diffraction data were collected at the General Medicine and Cancer Institute Collaborative Access Team (GM/CA-CAT) beamline at the Advanced Photon Source (Argonne National Laboratory, Argonne, IL) and were processed with the computer program XDS^28^. Diffraction data at the Se absorption edge was collected from the SeMet-TSP4-N_335_ crystals grown in the LiSO_4_. Phases were calculated by the single wavelength anomalous dispersion method (SAD) using the computer program PHENIX AutoSol^29^. The initial polypeptide chain was built with PHENIX Autobuild^30^. The structures of other TSP4-N crystal forms were determined by molecular replacement with the program PHASER^31^, and the structures were refined using the program REFMAC5^32^ as implemented in CCP4^33^. Model building and modification was performed using the interactive computer graphics program COOT^34^ and figures were prepared with PyMOL (The PyMOL Molecular Graphics System, Version 1.2r3pre, Schrodinger, LLC).

## Supplementary Information

Supplementary Information.

## Data Availability

The coordinates and structure factors were deposited in the Protein Data Bank with accession numbers **7REJ**, 7**RFO** and **7RFV**.

## References

[1] Kutter EM, Skutt-Kakaria K, Blasdel B, El-Shibiny A, Castano A, Bryan D (2011). Characterization of a ViI-like phage specific to *Escherichia coli* O157:H7. Virol. J..

[2] Plattner M, Shneider MM, Arbatsky NP, Shashkov AS, Chizhov AO, Nazarov S (2019). Structure and function of the branched receptor-binding complex of bacteriophage CBA120. J. Mol. Biol..

[3] Adriaenssens EM, Ackermann H-W, Anany H, Blasdel B, Connerton IF, Goulding D (2012). A suggested new bacteriophage genus: "Viunalikevirus". Adv. Virol..

[4] Chen C, Bales P, Greenfield J, Heselpoth RD, Nelson DC, Herzberg O (2014). Crystal structure of ORF210 from *E. coli* O157:H1 phage CBA120 (TSP1), a putative tailspike protein. PLoS ONE.

[5] Greenfield J, Shang X, Luo H, Zhou Y, Heselpoth RD, Nelson DC (2019). Structure and tailspike glycosidase machinery of ORF212 from *E. coli* O157:H7 phage CBA120 (TSP3). Sci. Rep..

[6] Greenfield J, Shang X, Luo H, Zhou Y, Linden SB, Heselpoth RD (2020). Structure and function of bacteriophage CBA120 ORF211 (TSP2), the determinant of phage specificity towards *E. coli* O157:H7. Sci. Rep..

[7] Taylor NM, Prokhorov NS, Guerrero-Ferreira RC, Shneider MM, Browning C, Goldie KN (2016). Structure of the T4 baseplate and its function in triggering sheath contraction. Nature.

[8] Mitraki A, Papanikolopoulou K, Van Raaij MJ (2006). Natural triple beta-stranded fibrous folds. Adv. Protein Chem..

[9] Granell M, Namura M, Alvira S, Kanamaru S, van Raaij MJ (2017). Crystal structure of the carboxy-terminal region of the bacteriophage T4 proximal long tail fiber protein Gp34. Viruses.

[10] Stummeyer K, Dickmanns A, Muhlenhoff M, Gerardy-Schahn R, Ficner R (2005). Crystal structure of the polysialic acid-degrading endosialidase of bacteriophage K1F. Nat. Struct. Mol. Biol..

[11] Holm L, Sander C (1993). Protein structure comparison by alignment of distance matrices. J. Mol. Biol..

[12] Kostyuchenko VA, Navruzbekov GA, Kurochkina LP, Strelkov SV, Mesyanzhinov VV, Rossmann MG (1999). The structure of bacteriophage T4 gene product 9: the trigger for tail contraction. Structure.

[13] Yap ML, Klose T, Arisaka F, Speir JA, Veesler D, Fokine A (2016). Role of bacteriophage T4 baseplate in regulating assembly and infection. Proc. Natl. Acad. Sci. U S A.

[14] Leiman PG, Arisaka F, van Raaij MJ, Kostyuchenko VA, Aksyuk AA, Kanamaru S (2010). Morphogenesis of the T4 tail and tail fibers. Virol. J..

[15] Urusova DV, Kinsella RL, Salinas ND, Haurat MF, Feldman MF, Tolia NH (2019). The structure of Acinetobacter-secreted protease CpaA complexed with its chaperone CpaB reveals a novel mode of a T2SS chaperone-substrate interaction. J. Biol. Chem..

[16] Yu AC, Worrall LJ, Strynadka NC (2012). Structural insight into the bacterial mucinase StcE essential to adhesion and immune evasion during enterohemorrhagic *E. coli* infection. Structure.

[17] Prokhorov NS, Riccio C, Zdorovenko EL, Shneider MM, Browning C, Knirel YA (2017). Function of bacteriophage G7C esterase tailspike in host cell adsorption: Function of bacteriophage G7C esterase tailspike. Mol. Microbiol..

[18] Manna A, Zhao H, Wada J, Balagopalan L, Tagad HD, Appella E (2018). Cooperative assembly of a four-molecule signaling complex formed upon T cell antigen receptor activation. Proc. Natl. Acad. Sci. USA.

[19] Altschul SF, Madden TL, Schaffer AA, Zhang J, Zhang Z, Miller W (1997). Gapped BLAST and PSI-BLAST: A new generation of protein database search programs. Nucleic Acids Res..

[20] Soding J, Biegert A, Lupas AN (2005). The HHpred interactive server for protein homology detection and structure prediction. Nucleic Acids Res..

[21] Huang X, Miller W (1991). A time-efficient, linear-space local similarity algorithm. Adv. Appl. Math..

[22] Brown PH, Schuck P (2006). Macromolecular size-and-shape distributions by sedimentation velocity analytical ultracentrifugation. Biophys. J..

[23] Brautigam CA (2011). Using Lamm-Equation modeling of sedimentation velocity data to determine the kinetic and thermodynamic properties of macromolecular interactions. Methods.

[24] Vistica J, Dam J, Balbo A, Yikilmaz E, Mariuzza RA, Rouault TA (2004). Sedimentation equilibrium analysis of protein interactions with global implicit mass conservation constraints and systematic noise decomposition. Anal. Biochem..

[25] Laue TM, Shah BD, Ridgeway TM, Pelletier SL, Harding SE, Horton JC, Rowe AJ (1992). Computer-aided interpretation of sedimentation data for proteins. Analytical Ultracentrifugation in Biochemistry and Polymer Science.

[26] Ortega A, Amoros D, Garcia de la Torre J (2011). Prediction of hydrodynamic and other solution properties of rigid proteins from atomic- and residue-level models. Biophys. J..

[27] Brautigam CA (2015). Calculations and Publication-Quality Illustrations for Analytical Ultracentrifugation Data. Methods Enzymol..

[28] Kabsch W (2010). XDS. Acta Crystallogr. D.

[29] Terwilliger TC, Adams PD, Read RJ, McCoy AJ, Moriarty NW, Grosse-Kunstleve RW (2009). Decision-making in structure solution using Bayesian estimates of map quality: The PHENIX AutoSol wizard. Acta Crystallogr. D.

[30] Terwilliger TC, Grosse-Kunstleve RW, Afonine PV, Moriarty NW, Zwart PH, Hung LW (2008). Iterative model building, structure refinement and density modification with the PHENIX AutoBuild wizard. Acta Crystallogr. D.

[31] McCoy AJ, Grosse-Kunstleve RW, Adams PD, Winn MD, Storoni LC, Read RJ (2007). Phaser crystallographic software. J. Appl. Crystallogr..

[32] Murshudov GN, Skubak P, Lebedev AA, Pannu NS, Steiner RA, Nicholls RA (2011). REFMAC5 for the refinement of macromolecular crystal structures. Acta Crystallogr. D.

[33] Winn MD, Ballard CC, Cowtan KD, Dodson EJ, Emsley P, Evans PR (2011). Overview of the CCP4 suite and current developments. Acta Crystallogr. D.

[34] Emsley P, Lohkamp B, Scott WG, Cowtan K (2010). Features and development of coot. Acta Crystallogr. D..

