# Supplementary material for: Structure of Escherichia coli O157:H7 bacteriophage CBA120 tailspike protein 4 baseplate anchor and tailspike assembly domains (TSP4-N)
Source: Sci Rep. 2022 Feb 8;12:2061. doi: 10.1038/s41598-022-06073-2 (PMC8825819; doi:10.1038/s41598-022-06073-2)
Supplement: Supplementary file 1 — Supplementary Information. [file 41598_2022_6073_MOESM1_ESM.docx]

**Supplementary Information**

**Structure of** *Escherichia coli* O157:H7 **bacteriophage CBA120 tailspike protein 4 baseplate anchor and tailspike assembly domains (TSP4-N)**

Kinlin L. Chao^1^, Xiaoran Shang^1^, Julia Greenfield^1,2^, Sara B. Linden^1^, Adit B. Alreja^1^, Daniel C. Nelson^1,3,*^ and Osnat Herzberg^1,2,*^

**The purified protein**

SDS-PAGE analysis of TSP4-N_335_ eluted from a Ni-Sepharose affinity column with 100 mM imidazole showed that it had molecular mass (MW_app_) of ~37 kDa, consistent with the calculated molecular mass (MW_calc_) of a 36.3 kDa monomer. The TSP4-N_335_ fraction eluted at 150 mM imidazole revealed a major band with MW_app_ >250 kDa, and a faint ~150 kDa band. All three bands were confirmed as TSP4-N_335_ by Western blotting analysis with C-terminal penta-His specific monoclonal antibodies Figure (S1A&B). SEC analysis revealed that TSP4-N_335_ from the 100 mM imidazole fraction eluted as a ~63 kDa, and from the 150 mM imidazole fraction as a ~470 kDa protein (Figure S1C), when compared to the globular protein standards. These values are higher than the MW_calc_ of 37 kDa for a monomer, 109 kDa for a trimer and 222 kDa for a hexamer, indicating higher Stokes radius and elongated molecular shapes.

**TSP4-N interaction with TSP1**

The SV experiments showed that TSP1 homotrimers sedimented as a 9.6 S species, consistent with the estimated S*_20,w_* of 10.2 S of the crystal structure (Figure 6A, Table 3). The SV analysis of a TSP1 and TSP4-N_335_ mixture at approximately an equimolar ratio showed a free TSP4-N_335_ hexamer peak with S*_20,w_* of 7.7 S, and two new peaks with S*_20,w_* of 11.4 S and 14.3 S that correspond to binding of one and two TSP1 trimers to the TSP4-N_335_ hexamer, respectively (Figure 6B, Table 4). At excess TSP1, the free TSP4-N_335_ peak disappears and the majority of the mixture exists as a 6:6 TSP1:TSP4-N_335_ complex (Figure 6B). The 3:6 and 6:6 TSP1:TSP4-N_335_ complexes appear to be elongated, with a higher *f/f_0_* of 1.8 when compared to that of TSP1 or TSP4-N_335_ hexamer alone (Table 3). The LEq analysis of the SV data was best fitted with *K*_d_ = 0.06±0.06 μM, *k*_off_ = 10^-4^ sec^-1^, *s*AB = 12.0 S, and *s*ABB = 15.1 S, using a two-site heterogeneous association model [A+B+B⮀AB+B⮀ABB], where A and B correspond to TSP4-N_335_ hexamer and TSP1 trimer, respectively (Figure 6B insert).

We also examined whether the D1-D2 head region of TSP4 affected TSP1:TSP4-N association using the TSP4-N_490_ protein. Free TSP4-N_490_ hexamers sedimented as an 8.8 S species (Figure 5B, Table 3). The *c*(*s*) distribution profile of an equimolar TSP1:TSP4-N_490_ mixture showed two peaks with higher S*_20,w_* values of 12.4 S and 15.7 S, in addition to a minor TSP1 peak (9.5 S) (Figure S3A, Table 3). Similar to the TSP1:TSP4-N_335_ complexes, the 12.4 S and 15.7 S species correspond to 3:6 and 6:6 TSP1:TSP4-N490 complexes, respectively. The LEq modeling of TSP1:TSP4-N_490_ SV data using the two-site heterogeneous association model was best fitted with *K*_d_ = 0.08 μM, *k*_off_ = 10^-4^ sec^-1^, *s*AB = 12.3 S, and *s*ABB = 16.8 S. The similar binding parameters and stoichiometry of TSP1 with either TSP4-N_335_ or TSP4-N_490_ demonstrates that the TSP4 D1-D2 head does not block the TSP1:TSP4-N association.

The TSP4-N_253_ fragment containing the AD-XD1-XD2 domains but lacking the XD3 domain sedimented at S*_20,w_* of 6.9 S (Figure 5C, Table 3). The SV profile of a TSP1 and TSP4-N_253_ mixture exhibits 7.0 S and 9.8 S peaks (Figure 6F), corresponding only to the free proteins (Figure 5C and 6A). Similarly, the TSP4-N_181_ fragment, containing only the AD-XD1 domains, sedimented at S*_20,w_* of 6.1 S (Figure 5D, Table 3). The SV analysis of the TSP1 and TSP4-N_181_ mixture revealed only peaks corresponding to the free proteins.

TSP1-N_166_ and TSP1-N_14-166_, comprising the D1-D2 domains and the ensuing α-helical neck region with and without the 13 N-terminal amino acids, tested the region of TSP1 that binds to TSP4-N. The addition of Zn^2+^ promoted the trimerization of the TSP1 head region. The SV analyses showed the presence of 1.7 S monomeric and 3.7 S trimeric species (Figure 6D), consistent with the calculated S*_20,w_* values for the monomeric and trimeric TSP1 head as seen in the crystal structure (1.8 S and 4.0 S, respectively). The SV analysis of the TSP1-N_14-166_ and TSP4-N_335_ mixture in the presence of Zn^2+^ showed a predominant 8.4 S species along with minor 4.9 S and 11.3 S peaks (Figure 6E). We attribute the 8.4 S and 11.3 S species to 3:6 and 6:6 TSP1-N_14-166_:TSP4-N_335_ complexes. The 4.9 S peak might represent aggregates of free TSP1-N_14-166_.

**TSP4-N interaction with TSP2**

The SV experiments showed TSP2 as homotrimers with S*_20,w_* of 9.9 S (Figure 7A, Table 3). By comparison, the calculated S*_20,w_* based on the TSP2 crystal structure lacking the 168 N-terminal amino acids is 10 S [1]. The *c*(*s*) distribution profiles of TSP4-N_335_ hexamer with increasing concentrations of TSP2 exhibited two new peaks with S*_20,w_* of 12.9 S and 15.0 S, in addition to the free TSP2 (9.9 S) and TSP4-N_335_ (7.7 S) species (Figure 7B, Table 4). We attribute the 12.9 S and 15.0 S peaks to TSP2:TSP4-N_335_ complexes with 3:6 and 6:6 stoichiometry, respectively. The LEq modeling of the TSP2:TSP4-N_335_ SV data assuming a two-site heterogeneous association model gave *K*_d_ = 0.11±0.8 μM, *s*AB = 12.0 S, *s*ABB = 16.7 S, and *k*_off_ = 2.5x10^-4^ sec^-1^. The global fitting of the TSP2:TSP4-N_335_ SE data gave *K*_d_ = 0.075 μM with the same model (Figure 7C). As with TSP1, the head region of TSP4 did not affect the TSP2:TSP4 association. The TSP2 and TSP4-N_490_ mixtures comprise a free TSP2 along with the 14.3 S and 16.7 S peaks, representing 3:6 and 6:6 TSP2:TSP4-N_490_ complexes, respectively (Figure S3B, Table 4).

The *c*(*s*) distribution profiles of TSP4-N_335_ in the presence of TSP2_89-921_ showed two peaks that represented the free proteins (Figure 7D). Similarly, TSP2_89-921_ also did not interact with TSP4-N_490_. However, the TSP2-N_179_ containing the XD2-XD3 domains and the N-terminal helix of D1 binds to TSP4-N_335_.

TSP2 binds to engineered TSP4-N proteins devoid of the XD3 domain (TSP4-N_253_) or the XD2-XD3 domains (TSP4-N_181_). The SV analyses showed two TSP2:TSP4-N_253_ complexes with S*_20,w_* values of 12.1 S and 14.8 S, and two TSP2:TSP4-N_181_ complexes with S*_20,w_* values of 12.0 S and 13.8 S (Figure 7E&F). In both cases, these new peaks may be attributed to complexes of 3:6 and 6:6 stoichiometry.

**The TSP1:TSP2:TSP4-N_335_ complex**

The *c*(*s*) distribution profile of equimolar TSP1, TSP2 and TSP4-N_335_ mixture showed four peaks; two minor peaks with S*_20,w_* of 10.3 S, 14.9 S, and two major peaks with S*_20,w_* of 17.4 S and 19.0 S (Figure 7G, solid line). The 10.3 S and 14.9 S peaks represent the free TSP2 and a 6:6 TSP2:TSP4-N_335_ complex (Figures 7A&B). The new 17.4 S and 19 S peaks are consistent with the formation of ternary TSP1:TSP2:TSP4-N_335_ complexes at 6:3:6 and 6:6:6 stoichiometry. At higher TSP2 concentrations, a higher amount of the 19 S ternary complex was formed compared with the 17.4 S species (Figure 7G). SV analyses of TSP1, TSP2 and TSP4-N_490_ mixture showed that the D1-D2 head domains of TSP4 did not impede the binding of TSP1 and TSP2. An equimolar mixture of these proteins showed peaks at S*_20,w_* of 9.9 S, 13.2 S, 16.4 S, 18.9 S and 21.1 S (Figure S3D). By analogy to the TSP1:TSP2:TSP4-N_335_ complex, the new 18.9 S and 21.1 S peaks are attributed to 6:3:6 and 6:6:6 TSP1:TSP2:TSP4-N_490_ complexes, respectively. The 13.2 S species may represent transient 3:6 TSP1:TSP4-N_490_ and TSP2:TSP4-N_490_ complexes, which did not resolve under the experimental conditions (Figures S3A&B).

**The TSP2:TSP3:TSP4-N_335_ complex – TSP3 does not bind directly to TSP4-***N*

TSP3 trimers undergo reversible oligomerization in solution, which was first detected during purification using a preparative SEC (Figure S4A). The majority of TSP3 eluted as the expected trimeric species, but a small amount eluted earlier, indicative of a higher oligomeric state. The SV analyses of the trimeric fraction indicated presence of both TSP3 trimers (S*_20,w_* = 8.4 S) and hexamers (S*_20,w_* = 12.9 S) (Figure S4B, Table 3). However, no TSP3 hexamers were formed in the presence of TSP1 and TSP2 as the SV of a TSP1-3 mixture displayed a broad peak with S*_20,w_* of 9.5 S, confirming that these proteins do not interact in the absence of TSP4-N (Figure 8A).

TSP3 did not associate with the TSP1:TSP4-N_335_ complex. The *c*(*s*) distribution profiles of TSP1, TSP3 and TSP4-N_335_ mixture showed 3 species. The 8.4 S peak corresponds to free TSP3. The two additional 10.9 S and 14.1 S peaks are consistent with the 3:6 and 6:6 TSP1:TSP4-N_335_ complexes, respectively (Figure 8C). Similar results were obtained with mixtures of TSP1, TSP3 and TSP4-N_490_.

The SV analysis of an equimolar TSP2, TSP3 and TSP4-N_335_ mixture showed three peaks with S*_20,w_* values of 9.9 S, 15.6 S and 19.3 S (Figure 8D). The 9.9 S peak represents the free TSP2 trimer. As shown in Figure 7B, the 3:6 and 6:6 TSP2:TSP4-N_335_ complexes have S*_20,w_* values of 12.9 S and 15.0 S, respectively. Therefore, the two new peaks at 15.6 S and 19.3 S are attributed to 3:3:6 and 6:6:6 TSP2:TSP3:TSP4-N_335_ complexes (Figure 8D, Tables 3-4). Similar increases in the S*_20,w_* values were observed when TSP3 binds to the TSP2:TSP4-N_490_ complex with the appearance of 16.4 S and 20.3 S peaks (Figure S3B and S3E).

A truncated TSP3 lacking the head, TSP3_156-627_, was used to test which TSP3 domain interacts with TSP2. As shown in Figure 8D, the TSP2:TSP3:TSP4-N_335_ complexes sedimented with S*_20,w_* of 15.6 S and 19.3 S. In contrast, the SV analyses of an equimolar TSP2, TSP3_156-627_ and TSP4-N_335_ mixture showed three peaks at 8.0 S, 10.2 S and 15.0 S, representing free TSP3_156-627_, free TSP2 and a TSP2:TSP4-N_335_ complex, respectively (Figure 8E). The absence of peaks corresponding to ternary complexes at 15.6 S or 19.3 S (Figure 8D) confirms that the TSP3 head is necessary for binding to the TSP4-N_335_:TSP2.

The SV experiments reveal that TSP3 binding requires the presence of TSP4-N XD3 domain (Figure 6B&F). A mixture of TSP2, TSP3 and TSP4-N_253_ lacking the XD3 domain shows three species: Free proteins (8.8 S and 10.0 S), and a 14.9 S species that corresponds to the TSP2:TSP4-N_253_ binary complex (Figure 8F). No peak can be attributed to a ternary complex.

**The quaternary TSPs complex**

The *c*(*s*) distribution profiles of an equimolar TSP1, TSP2, TSP3 and TSP4-N_335_ mixture showed two new peaks with higher S*_20,w_* of 19.8 S and 24.1 S (Figure 9A). Given that the largest ternary complex described above sedimented as ~19-19.3 S species, these new peaks represent quaternary TSP1:TSP2:TSP3:TSP4-N_335_ complexes, perhaps with stoichiometries of 6:6:3:6 and 6:6:6:6, respectively. Likewise, the SV analysis of the TSP1, TSP2, TSP3 and TSP4-N_490_ mixture revealed two new species at S*_20,w_* of 21.3 S and 24.9 S (Figure S3F), showing that as with the formation of the binary and ternary complexes, the TSP4 head did not interfere with the attachments of the TSP1-3 to TSP4-N.

**Modeling the TSP1-4 quaternary complex**

For the 3D model of the TSP1-4 complex, the TSP4-N model was produced by superposing the respective XD1 modules of TSP4-N and gp10 while keeping the same relative position of the TSP4 AD domain. Next, the TSP4 XD2 and XD3 modules were individually superposed on the respective modules of phage T4 gp10. The XD1-XD2 and XD2-XD3 linkers of TSP4-N are sufficiently long to maintain the covalent structure, however, they were built in arbitrary conformations and regularized within the computer program COOT because they differ in length from the gp10 linkers. This chosen domain arrangement allows the placement of the partner TSPs consistently with the available data, nevertheless, alternative orientations of the TSP4 XD2 and XD3 domains may exist, which would also satisfy the spatial constrains.

The full length TSP4 model includes the AD, XD1 and XD3 trimers as they appear in the two crystal structures of TSP4-N_335_. The XD2 trimer was taken from the crystal structure of TSP4-N_250_, where the three XD2 modules packed together. The published D1-D4 crystal structure of TSP4 (PDB accession number 5W6H; [2]) was added only after TSP1-3 were placed to avoid blocking of the binding sites. For the full length TSP2 model, homology models of the XD2 and XD3 domains were built using COOT, with the TSP4 XD3 domain as a template, because the two TSP2 domains share 25% and 28% sequence homology with TSP4 XD3, respectively. Energy minimization using Chiron relieved clashes [3]. The TSP1-3 trimers where then docked on the uninterrupted trimeric surfaces of the appropriated XD domains, in agreement with the AUC results as summarized in Figure 9B and illustrated in Figure 9C. Although not included in the model, the disordered 11 N-terminal amino acid residues of TSP1 [4], can be envisaged to extend into the inter-domain grooves of TSP4 XD3 trimer modules by analogy to the interactions of the N-terminal peptides of the short tail gp12 and the baseplate wedge protein gp11 with the respective XD2 and XD3 trimers of gp10 (PDB accession number 5IV5, [5]). Finally, the C-terminal D1-D4 domains of TSP4 were positioned approximately perpendicular to the 3-fold symmetry axis of the N-terminal AD-XD1 domains to obtain a structure resembling the negative-stained EM images [2].

**References**

[1] Greenfield J, Shang X, Luo H, Zhou Y, Linden SB, Heselpoth RD, et al. Structure and function of bacteriophage CBA120 ORF211 (TSP2), the determinant of phage specificity towards E. coli O157:H7. Sci Rep. 2020;10:15402.

[2] Plattner M, Shneider MM, Arbatsky NP, Shashkov AS, Chizhov AO, Nazarov S, et al. Structure and Function of the Branched Receptor-Binding Complex of Bacteriophage CBA120. Journal of molecular biology. 2019;431:3718-39.

[3] Ramachandran S, Kota P, Ding F, Dokholyan NV. Automated minimization of steric clashes in protein structures. Proteins. 2011;79:261-70.

[4] Chen C, Bales P, Greenfield J, Heselpoth RD, Nelson DC, Herzberg O. Crystal structure of ORF210 from E. coli O157:H1 phage CBA120 (TSP1), a putative tailspike protein. PLoS One. 2014;9:e93156.

[5] Taylor NM, Prokhorov NS, Guerrero-Ferreira RC, Shneider MM, Browning C, Goldie KN, et al. Structure of the T4 baseplate and its function in triggering sheath contraction. Nature. 2016;533:346-52.

**Supplementary Figures**


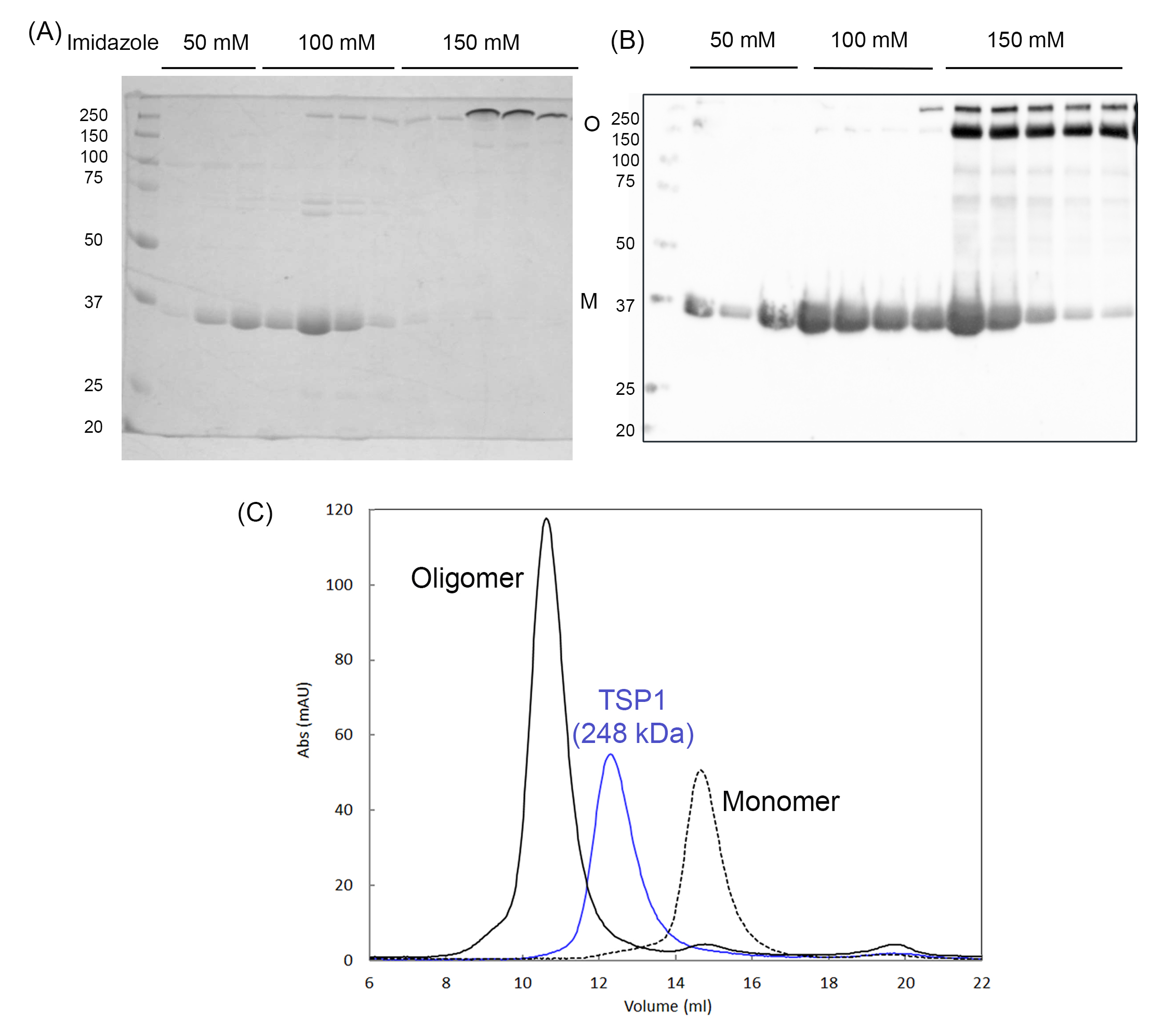


**Figure S1 -** Solution properties of recombinant TSP4-N_335_**. (A)** SDS-PAGE of the protein fractions eluted from a Ni-affinity column at 50, 100,150 μM imidazole buffer. **(B)** Western blotting of the gel using penta-His monoclonal antibodies. Gel imaged with BioRad ChemiDoc imager at auto exposure setting mode. (**C**) Elution profile of the monomeric (dashed line, 14.7 ml, MW_app_ ~63 kDa), oligomeric (solid line, 10.6 ml, MW_app_ ~470 kDa) TSP4-N_335_ and of TSP1 trimer used as a marker (blue line, 12.3 ml, MW_app_ ~204 kDa) from the analytical Superdex 200 column. Standard curve and void volume were calculated based on the elution volumes of blue dextran (2,000 kDa), horse spleen ferritin (440 kDa), bovine serum albumin (75 kDa), hen egg albumin (44 kDa), chymotrypsinogen A (25.6 kDa), bovine pancreas RNase A (13.7 kDa) and ATP (507 Da) in buffer containing 50 mM Tris (pH 8.0), 0.15 M NaCl, 0.5 mM EDTA and 0.02% NaN3 at 4⁰C.


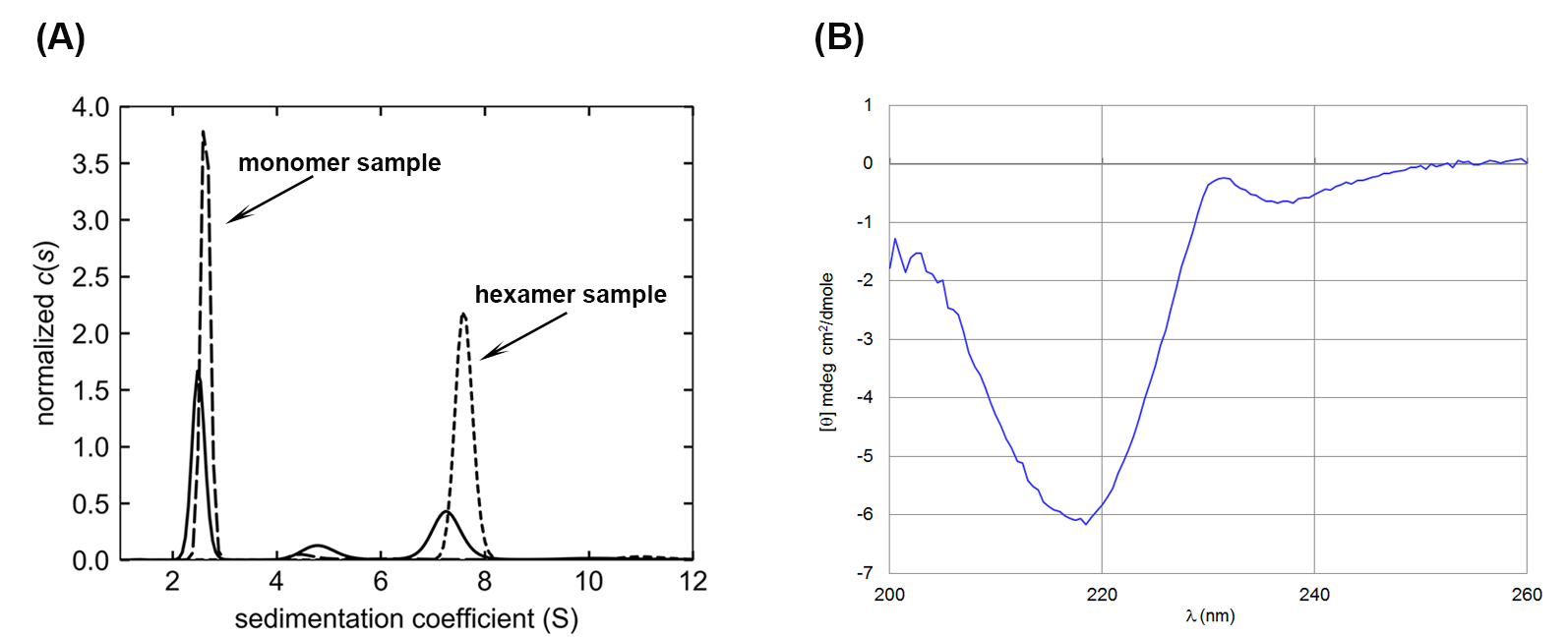


**Figure S2 –** Characterization of the monomeric TSP4-N_355_. (A) SV profiles of the 14 μM the monomeric fraction within a day after elution from the SEC column (2.7 S, long dash line), the 3.6 μM hexamer fraction (7.7 S, short dash line), and 10 μM monomeric fraction stored at 4⁰ C for a few weeks (solid line). The storage led to a slow partial conversion of the monomers into trimer (5.2 S) and hexamer forms. (B) Circular dichroism spectrum of the purified TSP4-N_335_ monomers prior to refrigeration. The protein exhibited a signature β-sheet profile with a minimum at 217 nm.

**
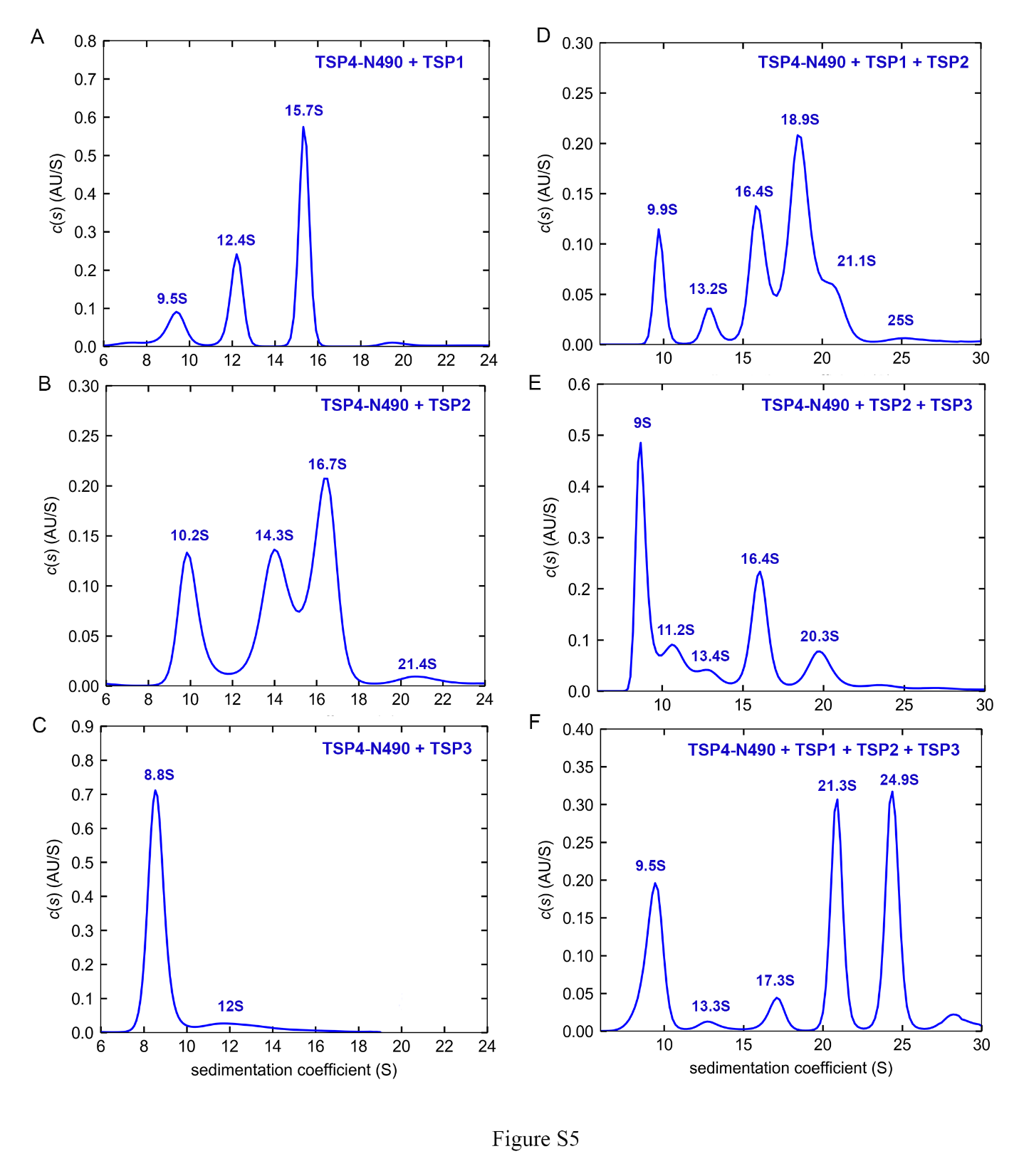
**

**Figure S3 -** SV analyses of the interaction between TSP4-N_490_ with TSP1, TSP2 and TSP3. The *c*(*s*) distribution profiles of **(A)** 4 μM each of TSP4-N_490_ and TSP1, (**B**) 4 μM each of TSP4-N_490_ and TSP2, (**C**) 5 μM each of TSP4-N_490_ and TSP3, (**D**) 3 μM each of TSP1, TSP2 and TSP4-N_490_. **(D**) 3 μM each of TSP4-N_490_, TSP1, andTSP3, (**E**) 3 μM each of TSP2, TSP3 and TSP4-N_490_ and (**F**) 2 μM each of TSP1, TSP2, TSP3 and TSP4-N_490_.


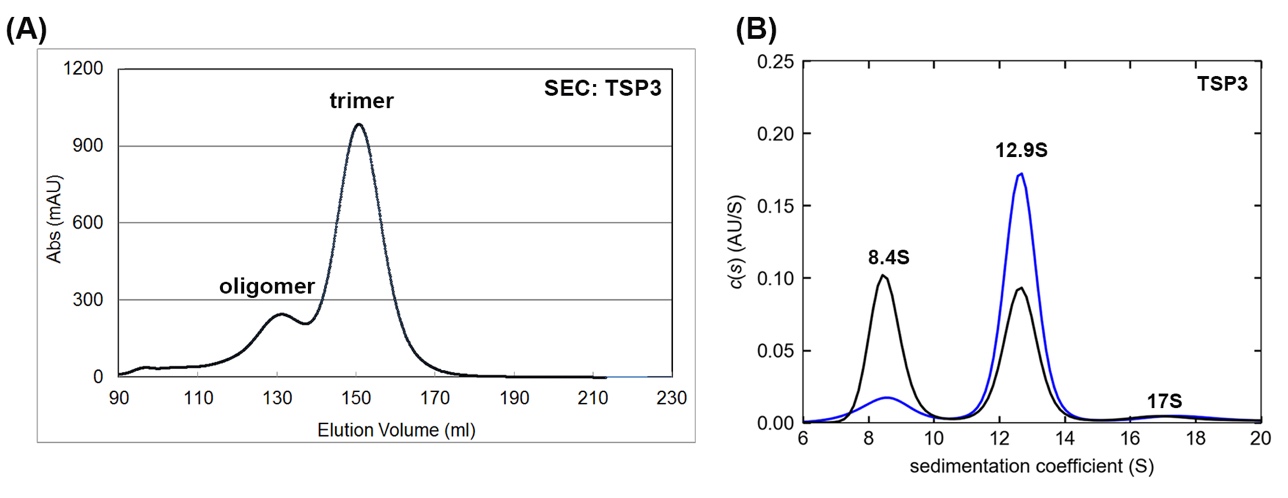


**Figure S4** - Solution properties of TSP3. (**A)** Elution of two TSP3 peaks from Sephacryl S-300 column at 132 ml and 151 ml. (**B**) The c(s) distribution profiles of TSP3 from 132 ml (1.5 μM, blue) and 151 ml (2.5 μM, black) peaks.
